# Air-STORM: Informed Decision Making to Improve the Success of Solar-Powered Air Quality Samplers in Challenging Environments

**DOI:** 10.3390/s25154798

**Published:** 2025-08-04

**Authors:** Kyan Kuo Shlipak, Julian Probsdorfer, Christian L’Orange

**Affiliations:** 1 Department of Mechanical Engineering, McCormick School of Engineering, Northwestern University, 633 Clark Street, Evanston, IL 60208, USA; 2 Department of Mechanical Engineering, Colorado State University, 1374 Campus Delivery, Fort Collins, CO 80523, USA; julian.probsdorfer@colostate.edu (J.P.); christian.lorange@colostate.edu (C.L.)

**Keywords:** air quality monitor, air pollution, low-cost sensors, numerical modeling, particulate matter, simulation, heat transfer, solar powered sensors

## Abstract

Outdoor air pollution poses a major global health risk, yet monitoring remains insufficient, especially in regions with limited infrastructure. Solar-powered monitors could allow for increased coverage in regions lacking robust connectivity. However, reliable sample collection can be challenging with these systems due to extreme temperatures and insufficient solar energy. Proper planning can help overcome these challenges. Air Sampler Solar and Thermal Optimization for Reliable Monitoring (Air-STORM) is an open-source tool that uses meteorological and solar radiation data to identify temperature and solar charging risks for air pollution monitors based on the target deployment area. The model was validated experimentally, and its utility was demonstrated through illustrative case studies. Air-STORM simulations can be customized for specific locations, seasons, and monitor configurations. This capability enables the early detection of potential sampling risks and provides opportunities to optimize monitor design, proactively mitigate temperature and power failures, and increase the likelihood of successful sample collection. Ultimately, improving sampling success will help increase the availability of high-quality outdoor air pollution data necessary to reduce global air pollution exposure.

## 1. Introduction

Air pollution is a major health hazard that contributes to premature death and disease worldwide, yet our understanding of global air remains incomplete due to significant gaps in data coverage and availability [1,2]. These gaps stem from the substantial expense and logistical challenges associated with achieving global coverage, including the need for extensive infrastructure, the difficulty of reaching geographically isolated regions, and the complexities of maintaining monitoring equipment in remote or resource-limited areas. Unfortunately, these barriers and resulting knowledge gaps disproportionately affect resource-limited regions and communities [3]. Incomplete air pollution data creates a knowledge bias, which can lead to research and policy that favors regions that have monitoring systems already in place [4,5,6]. This bias only worsens global gaps in air pollution data, further neglecting areas that most need additional monitoring.

Recently, there has been a significant increase in the availability and use of low-cost air pollution monitors [7]. These low-cost, low-power air pollution monitors have the potential to play a pivotal role in achieving truly global air quality monitoring. Their affordability makes it possible to deploy these monitors in greater numbers, substantially increasing coverage in areas that currently lack adequate monitoring infrastructure. Additionally, their low power requirements reduce restrictions on where they can be placed, allowing them to be deployed in remote or resource-limited locations that are otherwise inaccessible to traditional monitors. The International Energy Agency estimates that 737 million people still lack access to electricity [8], and the proportion of land-area without electricity is likely even greater. Due to inconsistent electricity access in many regions of the world [9,10,11], addressing gaps in global monitoring coverage will likely require sensor networks to rely on solar power systems. However, solar-powered air pollution monitors always require batteries for continuous operation, introducing additional challenges. These systems are particularly vulnerable to seasonal and periodic battery depletion, which can lead to power shortages and system failures [12,13]. Temperature fluctuations further complicate these issues, as both the monitors and their batteries are sensitive to thermal extremes. A wide range of air pollution sensors, including those used for particulate matter, gases (e.g., VOCs, ozone), and odors, exhibit performance degradation or drift when operating outside of optimal temperature ranges. When exposed to temperatures beyond their specified range, the lead-acid or lithium-ion batteries commonly used in these systems can suffer from reduced efficiency, safety risks, and shorter lifespans. Many systems incorporate battery management controls to mitigate these risks that prevent charging or discharging under extreme temperatures. These safeguards, however, can also result in complete system shutdowns if temperatures are not adequately regulated. As a result of these challenges, power interruptions and thermal stress have been widely documented as key factors limiting the reliability and data completeness of solar-powered air quality monitors [12,13].

Expanding air pollution monitoring networks in remote communities reliant on solar power is challenging, especially when deployments have reported data completeness rates below 50% [13,14,15,16]. By accounting for region-specific environmental conditions and constraints, monitoring systems can be optimized to improve reliability and performance ensuring that networks are equipped to operate effectively in even the most demanding settings. To address these challenges, we have developed an open-source tool called Air-STORM (Air Sampler Solar and Thermal Optimization for Reliable Monitoring) to predict enclosure temperatures and solar charging potential for outdoor air pollution monitors. This tool provides time-resolved temperature predictions using user-defined locations alongside a global database of temperature, solar radiation, and meteorology data. This model was designed not only to assess the feasibility of solar charging but also to proactively identify potential concerns, such as overheating, that could compromise performance. Successful deployment of solar-powered monitors does not happen by chance; it requires deliberate planning based on data-driven decisions and a clear understanding of environmental challenges. Through such intentional efforts, expanding monitoring into currently underserved regions becomes a more achievable and sustainable goal.

## 2. Materials and Methods

### 2.1. Model Overview

Air-STORM considers two major factors, both of which depend on incident solar radiation: solar charging potential and internal device temperature. It predicts the internal temperature of a user-specified instrument based on its dimensions, materials, heat generation from its components, deployment location, and time of year. It also predicts the ability of a monitor to run on solar power based on factors including local meteorology and sun position over time, device power consumption, battery capacity, and solar panel specifications. Both factors are essential for a solar-powered sampler to function properly, as sufficient solar charging and safe internal temperatures are necessary for reliable operation.

Air-STORM simulates a sampler running at a user-specified location to determine whether it can complete a “successful” sample, in which the instrument stays within the specified temperature bounds and maintains sufficient power to run uninterrupted. The tool is coded in Python3 and uses finite difference methods (FDM) to model heat transfer to the pollution monitor, which is represented as a three-dimensional rectangular enclosure consisting of six uniform plates. Forced convection is modeled using surface wind speeds and the rated air velocity from an internal cooling fan, and solar shield reradiation is modeled based on experimental correlations. Conceptual schematics of the modeling process are presented in SI Figures S7 and S8.

Model predictions are based on a combination of user-specified inputs and historical data. Air-STORM pulls historical solar radiation and meteorological data from NASA’s Prediction of Worldwide Energy Resources (POWER) API [17], using location and time to account for seasonal factors like cloud cover, sun position, and temperature. The POWER database offers geospatially resolved hourly solar and meteorological data from 2001 to the present, with meteorological variables provided at a 0.5° × 0.625° latitude by longitude resolution (approximately 55 km × 53 km) and solar radiation available at a 1° × 1° grid (approximately 110 km × 85 km). Though meteorology and radiation may differ within each grid-cell, variability can be accounted for by modeling multiple years of data, which will include substantial extremes that can encompass sub-grid cell differences (SI Figure S10). Higher-resolution datasets could also be integrated into the Air-STORM code base for specialized analysis, if necessary. The trade-offs of using these alternative data sources are discussed in the limitations section.

Presented below are key elements considered in the model. Further specifics on model validation, heat transfer equations, and computational methods are presented in detail in the SI.

(1) $$\frac{\partial }{\partial x}\left(k\frac{\partial T}{\partial x}\right)\;+\;\frac{\partial }{\partial y}\left(k\frac{\partial T}{\partial y}\right)+\;\frac{\partial }{\partial z}\left(k\frac{\partial T}{\partial z}\right)\;+\;\dot{q}\;=\;\rho c_{p}\frac{\partial T}{\partial t}$$

The heat diffusion equation (HDE, Equation (1)) describes transient heat conduction in any direction by applying the principle of conservation of energy, where $\dot{q}$ is the heat generation, ρ is the density, $c_{p}$ is the specific heat capacity, k is the thermal conductivity, and T is the temperature.

(2) $$T_{m}^{p+1}\;=\;Fo\left(T_{m+1}^{p}\;+\;T_{m-1}^{p}\right)\;+\;\left(1-2Fo\right)T_{m}^{p}\;$$

The finite difference method (FDM) discretizes the heat diffusion equation by dividing the material into individual points called nodes, each representing a section where temperature is computed. For one-dimensional heat transfer, FDM applies the fundamental heat diffusion equation at each interior conducting node (Equation (2)), solving for the temperature (T) at a timestep p+1 and node m, where Fo is the Fourier’s number, which characterizes the heat conduction rate. We can similarly discretize the HDE for surface nodes to numerically model free and forced convection, incident solar radiation, internal heat generation, and blackbody radiation. The Air-STORM tool uses four nodes based on sensitivity analyses (SI Figure S13) to balance accuracy with computational expense. We estimate internal air and battery surface temperatures using the area-weighted average temperature of the internal faces of the enclosure. The heat capacity of the air inside the enclosure is neglected because the enclosure and internal sampler components have substantially higher thermal mass (see Section 3.2). Plastics and metals typically have volumetric heat capacities over three orders of magnitude larger than air, making the heat capacitance of air insignificant.

To assess the validity of assuming that internal air temperature is equivalent to the average enclosure wall temperature, we conducted a targeted validation experiment. An ABS enclosure was outfitted with multiple K-type thermocouples to simultaneously measure: (1) the internal air temperature (suspended in the center of the enclosure), (2) the surface temperature on each inner wall, and (3) the battery surface temperature. These measurements were logged continuously under natural solar loading during the daytime. The enclosure was deployed in direct sunlight to simulate typical field conditions, where solar heating and battery performance are of primary concern.

Predicted temperatures depend on wind speed (SI Figure S15) and are sensitive to enclosure material characteristics, such as emissivity (SI Figure S17). Node count (SI Figure S13), initial temperature (SI Figure S14), and fan flow rate (SI Figure S12) were not found to have significant impacts on temperature predictions. For a given test, the thermal properties of enclosure materials were assumed to be constant (i.e., not temperature-dependent) and were obtained from the MatWeb and Engineering ToolBox external reference databases. The full fidelity FDM model was cycled on and off at a ratio of 1:3 with linear approximations to reduce computational expense (SI Figure S11).

(3) $$G\;=\;DNI\left[\sin\left(\gamma_{s}\right)\cos\left(\beta \right)\;+\;\cos\left(\gamma_{s}\right)\sin\left(\beta \right)\cos\left(\alpha_{s}\;-\;\alpha_{p}\right)\right]$$

(4) $$C^{p+1}\;=\;C^{p}\;+\;\left[G\;\times \;A_{s}\;\times \;\eta \;-\;P_{\mathrm{out}}\right]\Delta t$$

The incident radiation (G) on the solar panel is calculated using geometric relationships (Equation (3)) that account for the direct normal irradiance (DNI)—the solar radiation on the Earth’s surface perpendicular to solar rays, solar elevation ($\gamma_{s}$), solar panel tilt (β), solar azimuthal angle ($\alpha_{s}$), and solar panel azimuthal angle ($\alpha_{p}$). Solar power to the battery is approximated by multiplying the area of the solar panel, incident radiation (*G*), and solar panel efficiency (*η*), while the change in charge (C) is modeled to be the power influx from solar charging ($P_{\mathrm{in}}$) minus the sensor power consumption ($P_{\mathrm{out}}$), multiplied by the model timestep (Equation (4)). Detailed model descriptions can be found in the SI, and the full code is available on GitHub (version 1.0) [18] as a set of Python (version 3.8) scripts bundled with a Plotly Dash (version 2.7.1) application and graphical user interface (SI Figure S19). It should be noted that while we split solar panel sizing into area and efficiency to permit optimization of the two, typical solar panel wattage ratings are given assuming 1000 W/m^2^ of radiation, so the wattage can be found by multiplying the area and efficiency by 1000.

Model uncertainty predominantly comes from the uncertainty of the meteorological data used. Though the model is sensitive to material parameters chosen, these variations are substantially smaller than the modeled temperature and solar charging differences between days with complete cloud cover versus clear skies (SI Figures S11–S18). To estimate uncertainty propagation and meteorological variations when making predictions, the Air-STORM tool simulates temperatures using the 10% and 90% values of historical meteorological variables at each timestep, which we find to capture 97% of variability in simulated monitor temperatures (SI Figure S10).

### 2.2. Model Validation

#### 2.2.1. Temperature Validation Setup

Validation of the internal temperature model was conducted iteratively during model development, with a focus on assessing the reasonableness of key assumptions, such as the approximation that the internal air temperature closely tracks the enclosure surface and battery temperatures. Five enclosures of varying materials and geometries were tested on the rooftop of the Colorado State University Powerhouse Energy Campus. Each enclosure was equipped with one or more (depending on test) bare-end K-type thermocouples, affixed using a thermal bonding compound (OMEGABOND 300 Powder, Irlam, Manchester, UK), and logged using a Raspberry Pi 4 running a custom Python script. Thermocouples were placed on inner wall surfaces, (where applicable) battery surfaces, and at the center of the internal air space to compare temperatures.

The Raspberry Pi logged temperature data at intervals of 5 to 10 s, providing high-resolution thermal profiles. For reference environmental conditions, a meteorological station (Davis Instruments Vantage Pro2, Hayward, CA, USA) with an integrated solar radiation sensor recorded ambient temperature, relative humidity, and solar radiation every 5 min.

The enclosures tested included the following: Aluminum (7.6 cm × 7.6 cm × 7.6 cm), Fiberglass-reinforced polyester (FRP) (40.6 cm × 40.6 cm × 20.3 cm), Acrylonitrile butadiene styrene (ABS) (20.3 cm × 15.2 cm × 10.2 cm), a commercial PurpleAir enclosure (10.2 cm diameter PVC endcap, Draper, UT, USA).

All enclosures were placed on a flat sampling platform that allowed for unobstructed sun exposure for most of each day and positioned within 1 m of the meteorological station. Data was collected during summer months to capture high solar loading and ambient temperatures, which represent the most thermally challenging conditions. Sampling was conducted for both clear and partly cloudy days, and results were used to iteratively refine the model’s structure and assumptions.

#### 2.2.2. Solar Charging Validation Setup

Solar charging model validation was conducted at the same rooftop location using a custom solar-powered monitoring system. A 20-watt solar panel (Solperk, FD-EP-230201-1688, Kansas City, KS, USA) was mounted horizontally and connected to a 120 Wh, 12 V lithium-ion battery (Dakota Lithium, Seattle, WA, USA) housed inside an enclosure. An INA219 current and voltage monitor (Adafruit, Brooklyn, NY, USA) was used to measure charging current and battery voltage, with data logged by a Raspberry Pi.

Solar validation testing started by exploring charging dynamics under varying solar conditions. Data were collected across days with differing degrees of cloud cover to assess the model’s ability to capture transient solar availability and charging behavior.

Following this, the model was further evaluated under real-world operating conditions by connecting the solar panel and battery to a functioning air sampler. Specifically, the system powered a CSU-developed Aerosol Mass and Optical Depth (AMOD) monitor to test the model’s ability to capture realistic charging and discharging behavior. This deployment lasted 12 consecutive days, providing a complete cycle of diurnal solar charging and sampler load dynamics.

**Figure 1:**
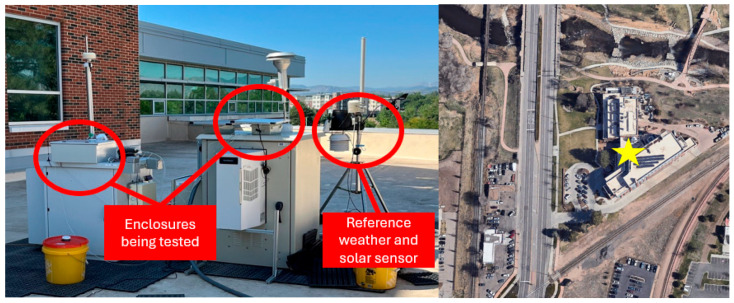
(**Left**) Experimental setup on the roof of the Colorado State University Powerhouse Energy Campus, used for local validation of air quality monitors and the Air-STORM tool. A weather monitoring unit was collocated to provide environmental reference data. (**Right**) Satellite image of sampling location. Star designates approximate sampling location.

A photograph of the rooftop sampling location and a satellite image of the validation site are shown in Figure 1 to provide context to the sample location.

### 2.3. Model Demonstration Methodology

Following model validation, we leveraged the Air-STORM tool’s predictive capabilities to explore the performance and design requirements of solar-powered air pollution monitors across diverse global environments. The demonstration case was based on seeking to achieve global coverage of a hypothetical, standard sampler setup with an ABS enclosure and 5 Watts of internal heat generation. Simulations were run for scenarios involving three common sampling configurations: a monitor with no solar shading, one with an attached solar shield, and a completely shaded monitor. In the first scenario, both the sampler and the solar panel are exposed to full sunlight during daylight hours. In the second, the solar panel covers the sampler while in full sun, causing it to absorb re-radiated heat. In the third, the solar panel is in direct sunlight during daylight hours, but the sampler is kept in complete shade. Battery charge predictions were made for a system consisting of a 0.5 m^2^ solar panel with zero tilt (i.e., parallel to the ground) and 18% efficiency, equivalent to a 90 W panel, and a 300 Wh battery with 80% efficiency, assuming an average sensor power consumption of 5 W. Though larger air quality monitors require more than 5 W, typical commercial low-cost sensors consume between 1 and 5 W (SI Table S1). Both temperature and charge predictions were made for the 2023 calendar year at a 2-degree geospatial resolution encompassing all non-Antarctica land areas. This fixed deployment configuration is not optimized for every location; its use here underscores the importance of tailoring system designs to local environmental conditions.

To establish upper and lower temperature limits for generic air samplers, we composed a list of 55 common air quality monitors (SI Table S1) and quantified the lower quartile maximum rated temperature, 45 °C, and the upper quartile minimum rated temperature, 0 °C (SI Figure S1). The upper limit of 45 °C corresponds to the maximum rated temperature for many lithium-ion batteries, beyond which they typically shut down, causing the samplers to lose power. The lower limit of 0 °C reflects the temperature at which many battery management systems restrict charging and discharging to protect the battery [19,20,21,22,23]. Although these thresholds were used in our demonstration case, they are not fixed and can be readily adjusted to reflect the specific thermal or operational limits of different batteries, sensors, or sampling systems. This adaptability allows the framework to be applied across a wide range of air quality monitoring platforms or alternative sampler constraints.

The demonstration case considers two failure types: (1) when temperatures fall outside acceptable thresholds and (2) when battery charge is depleted. These failures are quantified in two ways: the percentage of total time during failure conditions and the percentage of days on which any failure is recorded. The time-based measure applies to sensors that automatically resume sampling once temperatures are back within the acceptable range and batteries have sufficient charge. The day-based measure is relevant for sensors requiring manual restarting—where even brief failures can lead to hours or months of missing data. The model results assume samplers are always restarted when possible, representing a conservative case of maximum power consumption and heat generation.

### 2.4. Case Studies

To illustrate the variability of deployment challenges, we examined two contrasting case studies: New Delhi, India, and Montreal, Canada. New Delhi experiences high failure rates due to both extreme heat and seasonal drops in solar potential, especially during the monsoon season. In contrast, Montreal faces failure risks primarily from prolonged sub-zero temperatures that limit battery charging and periods of low solar potential during the winter. These examples highlight the need for location-specific design strategies and demonstrate the model’s utility in identifying failure periods.

## 3. Results and Discussion

### 3.1. Model Validity

To assess the validity of the Air-STORM monitor internal temperature prediction model, we compare predicted internal temperatures with experimental results. Monitor temperatures were simulated using co-located solar radiation and meteorology data, and temperatures were averaged to a 15 min resolution to address autocorrelation and correct temporal dependencies.

Performance metrics were calculated in Table 1 to evaluate the predictive accuracy of the model. A strong linear agreement was seen for all the enclosure types and sizes tested, with each coefficient of determination (R^2^) over 0.87, suggesting the Air-STORM model captured most observed temperature variations, and corresponding to a Pearson correlation coefficient (r) over 0.96 in each case. The root mean squared error (RMSE) ranged from 1.4 °C for the PVC enclosure to 3.5 °C for the aluminum enclosure. The simulated PVC enclosure internal temperatures had the best correlations and smallest error compared to observed temperatures. This is likely due to increased mixing from this enclosure being a PurpleAir monitor with an open-end, whereby PVC endcaps are used to house the sensor components. However, it also demonstrates the ability of the Air-STORM tool to accurately simulate temperatures for sensors of non-rectangular geometries.

Model error was found to increase with the conductivity of the enclosure material. The sensitivity of both the error and mean bias of predictive performance with material conductivity is related to the computational complexity of highly conductive materials. Conductive materials exhibit higher heat transfer rates and require smaller FDM time steps and step sizes to accurately capture thermal gradients (SI Equation (S8)). The simulated aluminum enclosure had the poorest correlation and highest error with observed temperatures, likely due to the increased complexity of modeling highly thermally conductive enclosures. It also exhibited the largest mean bias, with the Air-STORM tool underestimating temperatures by 1.89 °C. The bias between simulated and observed temperatures within the aluminum enclosure demonstrates a limitation of this model. The Air-STORM tool assumes no conduction radially between enclosure sides, which is a poorer assumption for more conductive materials and smaller enclosures. However, mean biases and RMSE between observed and predicted temperatures for the three plastic enclosures were under 1.1 °C and 2.7 °C, respectively, suggesting the Air-STORM tool, can accurately predict internal temperatures for monitors with a variety of plastic enclosure materials.

**Figure 2:**
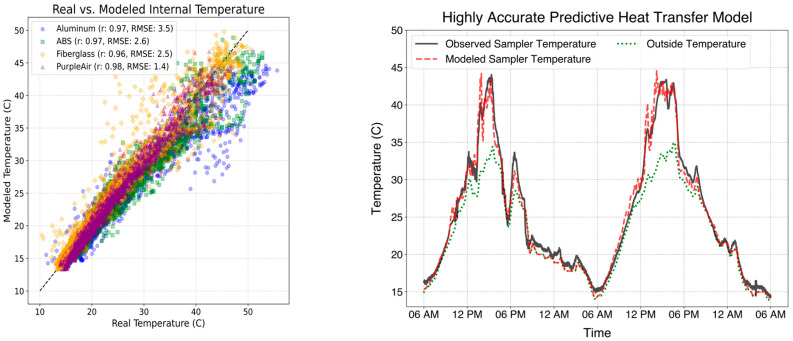
(**Left**): Modeled vs. observed internal temperatures for four enclosures over one month. Model deviation was calculated in terms of root mean square error (RMSE). (**Right**) Modeled vs. experimental time-series data of internal temperatures for a fiberglass-reinforced plastic (FRP) enclosure over 48 h. The figure compares modeled predictions (dashed red) with experimental internal (solid black) and outside temperatures (dotted green).

Figure 2 (left) shows a scatterplot of observed vs. simulated internal temperatures for the four enclosures of various sizes and shapes tested. Simulated temperatures exhibited the highest bias compared to observed temperatures during periods with the most variable solar radiation. In the absence of solar radiation, the internal monitor temperature typically approaches ambient temperatures because there is no temperature differential between the enclosure and outside air. For example, at night, simulated temperatures, ambient temperatures, and observed temperatures are nearly identical (Figure 2 (right)). Though model validation only extended for one month of continuous data collection, this period exhibited highly transient average diurnal DNI and ambient temperature profiles, in addition to day-to-day variation (SI Figures S20 and S21), making it a challenging environment for Air-STORM’s numerical heat transfer simulation, and a fitting validation period.

The model error is significantly smaller than the error if predicting internal sampler temperatures was conducted using ambient outdoor air temperatures (Figure 2 (right)). In particular, predicting the maximum daily temperature, which is most relevant for deploying sensors, using just ambient outdoor air temperatures results in mean errors and biases exceeding 10 °C. However, the daily maximum internal temperatures were accurately predicted for plastic enclosures with under 3 °C of mean absolute error (MAE), and under 1.2 °C MB. An uncertainty or error of 3 °C is unlikely to substantially impact decisions about monitor placement, as it is always advised to allow for some margin of safety when considering the operational limits of an instrument. This error is also smaller than the uncertainties related to internal heat generation (SI Figure S16), emissivity of enclosure materials (SI Figure S17), and probability of shading such as cloud cover (SI Figure S18).

Although more computational resources could be dedicated during analysis to reduce model error, this would increase computational time. Differences in solar shading due to imperfect collocation also contribute to the overall error. The validation enclosures and the reference solar sensor were spaced about 1.5 m apart, leading to slight differences in incident solar radiation (Figure 1). For example, instances where only the reference sensor or the sampler is shaded by partial obstructions can create slight discrepancies between modeled predictions and actual measurements. Although these discrepancies are real, they are minor in the context of predicting probable failures, as the analysis focuses on long-term performance over extended periods.

The accuracy of the solar charging model was similarly evaluated by comparing predicted charges with observed charges of a sampler operating on a battery with a solar panel co-located with the reference incident solar sensor (SI Figures S5 and S6). The simulated charge of the 120 Wh battery closely matched observations, with a mean bias of −1.63 Wh, root mean square error of 3.76 Wh, mean absolute error of 2.84 Wh, and coefficient of determination of 0.93. The worst model performance occurred when the solar panel was partially shaded. The 80 mm^2^ reference sensor would often measure either full sunlight or full shade, whereas the larger solar panel (0.18 m^2^) would be in partial sunlight. Despite these minor discrepancies, the overall trends are closely aligned. It should be noted that solar panel performance was evaluated to establish a charging efficiency of 16% before running the simulations.

Although model accuracy is important, Air-STORM’s value lies in its ability to assess the feasibility of running a sampler on solar power without exceeding maximum temperature thresholds over extended periods. Uncertainty exists in both model predictions and the meteorological information obtained from the POWER dataset. However, modeling with years of historical data allows users to predict when and how often failure will occur in a typical year. Combining this information with general engineering standards and safety factors enables the design and iteration of sampling systems for specific deployment needs.

### 3.2. Sensitivity Analysis

**Figure 3:**
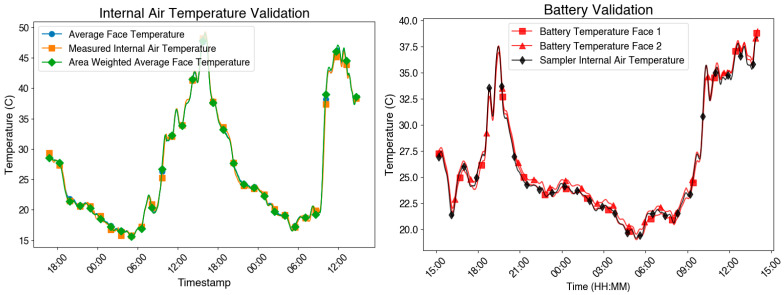
(**Left**) Comparison between enclosure inner surface temperature and internal air temperature. (**Right**) Comparison between internal air temperature and battery surface temperature. The minimal differences observed between surface and air temperatures support the use of simplified thermal assumptions in the model.

The results of the surface-temperature vs. air-temperature experiment shown in Figure 3 demonstrate that the internal air temperature, enclosure wall temperature, and battery surface temperature were nearly identical throughout the measurement period. The temperature differences among the three locations were consistently below 0.25 °C, even during peak solar loading. This supports the assumption used in the Air-STORM model that the internal air temperature equilibrates rapidly with enclosure surfaces and battery components.

To put this in perspective, the thermal equilibration time of air is governed primarily by the enclosure’s internal volume and the surface area available for heat exchange. In small sensor enclosures, the air has low thermal inertia (e.g., for 5 L of air, the heat capacity is ~6 J/K), and conduction and radiation from the surrounding surfaces rapidly bring it to thermal equilibrium. Based on standard convective heat transfer coefficients (~5–10 W/m^2^·K) and enclosure wall areas, the thermal response time of air in such enclosures is on the order of seconds to minutes.

Internal air temperatures only meaningfully diverge from surface temperatures to a degree such that spatial gradients become significant in large enclosures with poor air circulation. Since most air pollution enclosures are relatively small, the simplified approach remains valid. More complex modeling of air volume and internal convection would not appreciably improve predictive performance under the use cases examined here.

A sensitivity analysis was conducted to assess the influence of other modeling assumptions and input parameters on the predicted temperature and power outcomes. Key factors evaluated included the number of nodes used in the FDM calculations, the impact of external influences such as fan and wind speeds, and modeling reradiation from a solar shield. Additional considerations included averaging approaches for internal surface temperatures, assumed starting temperature, internal heat generation, and material emissivity. Among these, internal heat generation and material emissivity were found to have the most substantial impact on model predictions. Both parameters can be determined with relatively high accuracy for a given sampler, reducing uncertainty in real-world applications. Full details of the sensitivity analysis, including specific parameter variations and their effects, are provided in the Supplemental Information.

### 3.3. Model Demonstration

Designing a plan for a successful solar-powered air quality monitoring system must account for the specific environmental conditions of each location where monitors will be placed. Solar potential and the resulting internal monitor temperatures vary greatly by region and can lead to high failure rates in certain areas. Accurate and predictive modeling is essential to anticipate these challenges and minimize failures during sampler deployment. In some cases, extreme heat and intense solar radiation may render samplers unsuitable for specific locations, even if shading is used. To illustrate the variability of air quality monitor failures, a typical low-cost solar-powered sampler setup was modeled for 2023 at a 2° × 2° resolution across all non-Antarctica land areas. While the maps presented reflect simulations for a single year, the model can analyze data over any desired period, providing valuable insights for long-term planning.

**Figure 4:**
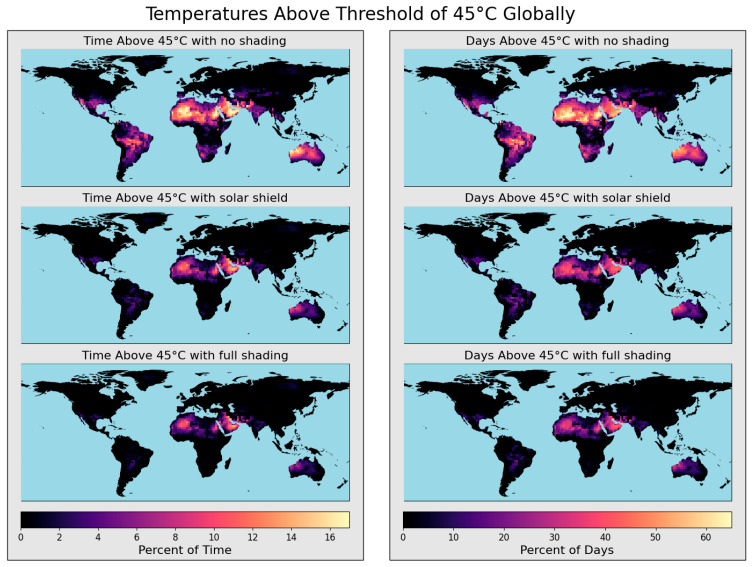
Global modeling of temperature-based overheating failure for 2023. Failure is characterized in two ways: the percentage of time internal temperatures exceed 45 °C (**left** column) and the percentage of days when internal temperatures exceed 45 °C at least once (**right** column). Three shading conditions are evaluated: no shade (first row), shade with a solar shield (second row), and full shade (third row).

Monitors without solar shading experienced failure during >5% of the total modeled duration in many regions, including South America, southern North America, the Sahara, southern Africa, the Arabian Peninsula, South Asia, Southeast Asia, and Australia (Figure 4). In these areas, the frequency of days with temperature exceedances was also high. Some locations surpassed the maximum rated internal temperatures on over 60% of days in 2023. Even failure rates as low as a few percent can significantly impact long-term monitoring. Temperature-based failures are more likely to occur during the day, leading to under-sampling in the afternoon. This can introduce data bias and affect the representativeness of the samples, especially if emission sources follow strong diurnal patterns. Examples include traffic emissions, evening cooking activities, and pollution changes driven by atmospheric mixing layer variations due to temperature shifts. For samplers that require manual restarting to resume operation, remote locations with difficult access are of particular concern, as a single failure could result in weeks or months of lost data.

Equipping monitors with a solar shield, such as a solar panel above the sampler, substantially reduced temperature-based failure rates by limiting incident solar radiation to the enclosures. The area-weighted average percentage of time spent above 45 °C for the global domain was 2.6% with no shade, 1.0% with solar shielding, and 0.7% with full shade. The average percentage of days with temperatures simulated above 45 °C at least once was 11.8% with no shade, 4.7% with solar shielding, and 3.2% with full shade. Solar shields were especially effective in the Americas, eastern Australia, sub-Saharan Africa, and Southeast Asia, reducing failure rates in some areas from over 10% of the total time to below 5%. Area-weighted averages are used because due to the latitude–longitude gridding of the domain of the Mercator projection, grid-cells at higher latitudes have smaller areas than tropical grid-cells.

Predictions from the Air-STORM model indicate that much of the world would likely experience temperature exceedance failures at least once during the modeled year, even if failures were infrequent. 69.2% of areas with no shading, 43.4% of areas with solar shielding, and 35.6% of areas with full shading were simulated to have temperatures surpass 45 °C, presenting a substantial fraction of potential sites for sampler deployments. The 25.8% reduction in temperature failures with solar shielding highlights the efficacy of solar shields to mitigate temperature threshold exceedances. Full shading was shown to further reduce high-temperature-based failures, but the incremental benefits of full shading in comparison to the use of only a solar shield were found to be minor in most regions. These results underscore the significant impact of solar radiation on internal temperatures.

In some regions, temperature concerns exist even if solar radiation is fully removed. For example, monitor temperatures in regions such as Saharan Africa, the Arabian Peninsula, the Persian Gulf, and Western Australia were still modeled exceed 45 °C on more than 30% of days and 10% of the total time with full shading. In these areas, monitors would require active cooling systems or specially rated batteries and power systems to operate reliably under such extreme conditions.

Sampling campaigns’ plans must also account for low temperatures. Sensor networks can encounter battery performance issues when internal temperatures drop below the minimum operating limits. Consumer-grade lithium-ion batteries cannot be safely charged at sub-zero temperatures due to the formation of metallic lithium on the anode, which can degrade battery performance and pose safety risks [24]. To ensure reliable operation, it is often recommended that batteries are only charged at temperatures above freezing, which can limit when solar-powered systems can be used in some regions.

**Figure 5:**
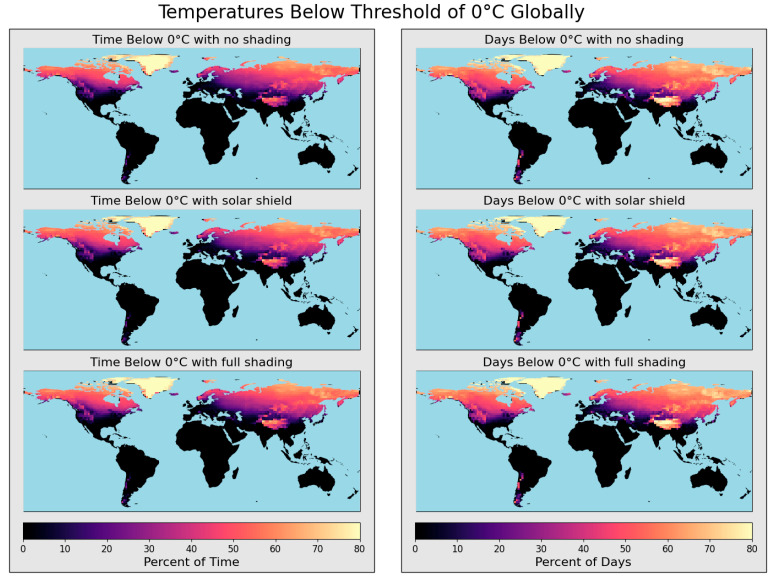
Global modeling of low-temperature failure for 2023. Failure is characterized in two ways: the percentage of time internal temperatures fall below 0 °C (**left** column) and the percentage of days when internal temperatures fall below 0 °C at least once (**right** column). Three shading conditions are evaluated: no shade (first row), shade with a solar shield (second row), and full shade (third row).

Figure 5 shows simulated failure rates due to battery freezing for the same hypothetical monitor enclosure used in overheating scenarios. In this case, shade would be detrimental to sampling success, as solar radiation can help prevent batteries from reaching sub-zero temperatures. As before, two failure modes are considered: the percentage of time the battery is at sub-zero temperatures and the percentage of days during which batteries reach sub-zero temperatures at least once. The two modes of simulated failure rates for temperatures below zero are much more similar than when considering overheating. Peak solar potential windows are narrow, leading monitors in warmer climates to overheat for short periods, whereas if monitors reach minimum temperature thresholds, they typically dip below for extended hours of the day.

Failure rates of battery temperatures dropping below zero were generally higher at higher latitudes, with topographical exceptions in locations at altitude, which are typically much cooler than at latitudinally adjacent locations at sea level. Greenland was modeled to have internal battery temperatures below 0 °C year-round, with northern territories of North America and Eurasia reaching similar failure rates. Failure rates were elevated at high altitudes, including in the Andes Mountains, Swiss Alps, Rocky Mountains, Caucus Mountains, Tibetan Plateau, and the Tian Shan ranges.

For each mode of failure and shade case, 46.3% of areas experienced sub-zero battery temperatures at least once during the simulated year. Notably, solar shielding had minimal effects on the average percentage of days spent below zero for the global domain (no shade: 18.0%, solar shield: 18.1%, full shade 18.1%) while it only had slightly higher impacts on the average percent of time spent freezing (no shade: 13.8%, solar shield: 14.2%, full shade 14.4%). Such a small effect suggests that solar shielding is more significant as a solution to mitigate battery overheating than as a contributor to battery freezing. Additionally, monitor failure rates were sensitive to heat generation from the circuitry and enclosure insulation. To size a heater, users could simply model higher heat generation rates, which would increase battery temperatures, and to model insulation, users can provide the thickness and thermal properties of the insulation rather than the enclosure, as the insulation will dominate heat transfer behavior.

Similarly, solar charging was modeled globally to identify high-risk regions for solar-powered air quality monitors and demonstrate the utility of Air-STORM. A solar-powered system consisting of a 0.5 m^2^ panel with 18% efficiency, a 300 Wh battery with 80% storage efficiency, and a panel at 0 degrees tilt was used for all modeling scenarios. A single configuration was used for all regions to demonstrate the striking variabilities in power failure rate risk for a given, completely unoptimized system. As in previous sections, two failure modes were examined: the percentage of time the battery was discharged and the percentage of days during which the battery was discharged at least once. While a general trend of reduced solar power efficacy farther from the equator was observed, other non-intuitive factors, such as monsoon and hurricane seasons, coastal meteorological phenomena, and anthropogenic air pollution, also substantially impacted failure rates over time.

**Figure 6:**
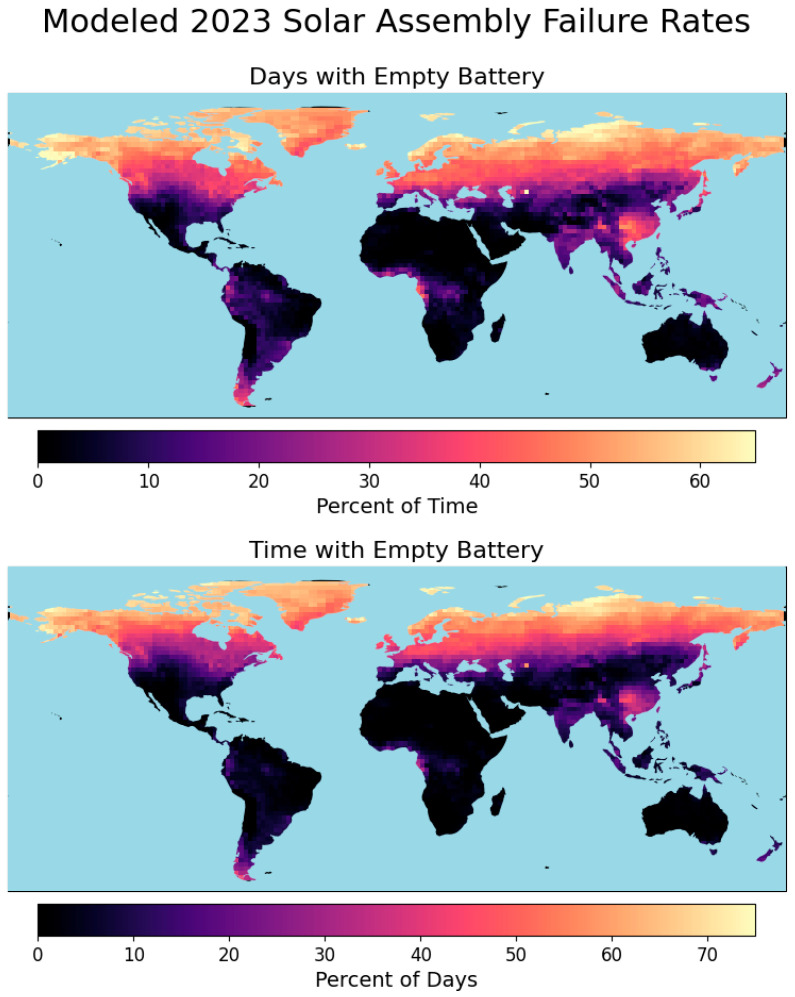
Global modeling of solar energy-based failure for 2023. Failure is represented in two ways: the percentage of time the battery is empty (**top**) and the percentage of days when the battery is empty at least once (**bottom**).

The highest failure rates were observed in the northernmost latitudes of North America and Eurasia due to reduced solar potentials, where batteries were modeled to be empty over 60% of the time and on 70% of days (Figure 6). These areas are also influenced by having optimal solar elevation angles far from zero, further reducing solar power. Similarly, high failure rates were noted at Cape Horn in South America, the southernmost region studied. The two failure modes evaluated exhibited similar failure rates. Solar potential typically peaks for only a few hours a day, leading monitors to overheat for shorter periods while under the highest solar radiation before they cool down. Conversely, discharged batteries of solar modules will remain empty for hours or even days while solar potentials are low until periods of high solar radiation recharge batteries and can meet sampler demands.

In addition to regions farthest from the equator, tropical regions, including central South America (excluding the Andes), west-central Africa, India, and Southeast Asia, were found to have power concerns on over 20% of days, although for much smaller percentages of the time. These regions benefit from high clear-sky DNI throughout the year, but incident solar radiation is highly inconsistent due to factors such as high humidity and frequent cloud cover, especially during monsoon and hurricane seasons. A notable hotspot in battery failure rates was identified in East China, likely resulting from high levels of air pollution, which can significantly reduce incident solar radiation through scattering and absorbing radiation [25,26,27,28].

Power and temperature failures were modeled separately, but in practice, those deploying monitors are likely most concerned with the overall risk of failure. When combining the temperature-related and solar-energy battery failure rates, it becomes evident that at least one of these issues will be of concern when deploying air quality monitors with solar energy systems, regardless of the monitoring location. In some tropical, warm-climate regions, there is a risk of failure due to both temperature exceedances and insufficient solar charging. New Delhi India was selected as a case study due to the significant challenges for sensor deployment from both temperature and solar-charging concerns. Here, we analyze annual and diurnal failure profiles to investigate potential design solutions.

### 3.4. New Delhi Case Study

**Figure 7:**
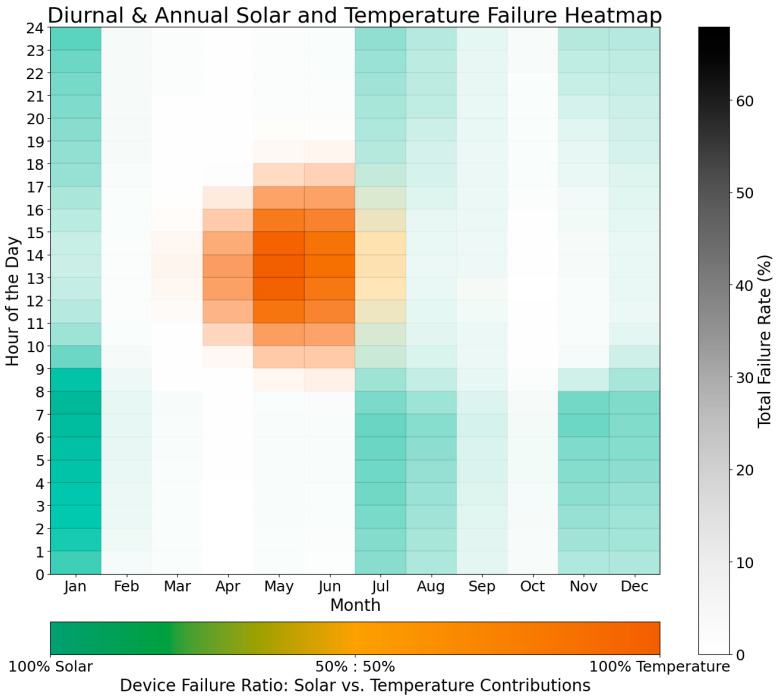
Heatmap of monthly and diurnal failure rates for the predictive temperature and solar charging models in New Delhi, India, over a 10-year period (2014–2023). Color intensity indicates the total failure rate, while color pigment distinguishes failure types: temperature exceedances (orange) and battery depletion (teal).

The heat map in Figure 7 illustrates monthly and diurnal failure rates predicted for a standard monitor, identical to those previously discussed, over a 10-year period (2014–2023). Color intensity represents overall failure rates, while color pigment differentiates failure causes: orange indicates temperature exceedances, and teal represents battery depletion due to insufficient solar charging. Temperature-related failures were concentrated in the months of April to June, when high ambient temperatures and intense solar radiation were likely to cause temperature concerns. Despite high ambient outdoor air temperatures, these failures were less frequent during the summer months of July and August due to reduced solar radiation during the monsoon season. Diurnally, temperature failures occurred almost exclusively during peak daylight hours (10:00–16:00), when peak outdoor temperatures coincided with maximum direct normal irradiance (DNI). Isolated temperature and solar failure heatmaps are shown in SI Figures S2 and S3.

As expected, insufficient solar potential for battery charging was concentrated in November, December, and January due to reduced daylight hours and lower solar radiation. However, failures of similar frequency were also observed during July, August, and parts of September, coinciding with the monsoon season. During monsoon months, extensive cloud cover and high humidity drastically reduce the amount of solar radiation available for battery charging. This finding is counterintuitive because summer is conventionally associated with high solar potential. Solar failures were most frequent at night and in the early morning, when the power drawn from the sampler depleted the energy stored during daylight hours. However, seasonal variations in solar potential had a stronger influence on failure rates than diurnal patterns, emphasizing the importance of considering annual trends when designing solar systems.

Regions such as New Delhi present significant challenges for air quality monitoring due to the dual risks of high temperatures and limited solar potential, which persist year-round. Only three months: February, March, and October, were predicted to have average total failure rates below 10% at each hour of the day. Highlighting these risks does not suggest that sampling cannot be successfully conducted in these areas, but emphasizes the need to tailor solutions to the specific regions where deployments are planned. These failure patterns highlight the importance of optimized system designs to address both temperature- and solar-related challenges. Predictive models using historical weather data can uncover counterintuitive failure trends and guide the optimization of monitoring systems to prevent data losses during critical seasons and times of interest.

In addition to the New Delhi case study, an illustrative analysis was also conducted for Montreal, Canada, a region with vastly different temperature conditions (SI Figure S3). While both solar potential and temperature remain key concerns, the primary failure risk in this case is from prolonged exposure to extreme cold rather than excessive heat. This contrasts with the challenges observed in New Delhi, where overheating and seasonal reductions in solar radiation were the dominant issues. Despite these differences between New Delhi and Montreal, the model identified the periods of the year with the highest failure risk in both locations, demonstrating its adaptability across diverse environments. The mitigation strategies required for these two cases differ significantly, highlighting the value of predictive modeling in informing location-specific deployment decisions. Details of the Montreal case study are provided in the Supplemental Information.

Informed by the model developed in this study, monitoring campaigns can implement targeted measures to maximize efficiency and ensure reliable sampling. Such strategies include shading sensors in warm climates, sizing panels and batteries to balance performance and cost, optimizing panel orientation to maximize solar potential during high-priority seasons and times for data collection, and heating or insulating monitors in cold locations.

### 3.5. Model Limitations

While the Air-STORM tool can provide valuable insight into the feasibility and challenges of solar-powered air pollution monitoring, several limitations should be acknowledged. First, the model relies on historical meteorological data available at hourly resolution and limited spatial resolutions. In contrast, the validation of the model was conducted using 5 min resolution experimental data. The coarser data used for predictive modeling does not account for short-term fluctuations in solar radiation. This discrepancy can introduce uncertainties, particularly in regions with highly variable cloud cover or rapid weather shifts. Additionally, while the tool accounts for major thermal and power constraints, other environmental factors, such as dust accumulation on solar panels, humidity effects on electronics, and potential sensor degradation, are not explicitly modeled. FDM modeling was chosen for its small computational expense to enable multiple, quick simulations of multi-year periods. However, the accuracy of the assumptions associated with 1D FDM modeling decreases when simulating smaller monitors and higher thermal conductivities. In the Air-STORM application, shading from obstructions is currently modeled to occur at fixed times, but shading times will change over the course of the year, introducing slight bias into shading approximations. Battery aging is not explicitly modeled; however, users are encouraged to assume a conservative, end-of-life battery storage efficiency when designing deployments. Lithium-ion batteries can experience a 10–30% reduction in capacity over 2–5 years, depending on use conditions, especially under sustained high temperatures or deep discharge cycles [29]. Incorporating this degradation into sizing decisions can help maintain reliability throughout the device’s operational lifespan. Lastly, the model demonstration section of this study used a typical low-power example air sampler; however, the model is structured to allow modeling a wide range of device powers. A key consideration when running higher-power devices is accurately estimating the proportion of power input converted to heat, as this relationship changes with scale. To model a higher-powered monitor in the Air-STORM tool, the internal heat generation would likely increase (SI Figure S16), and the battery and solar panel would need to be sized to accommodate the larger power consumption (SI Figure S22). Despite these limitations, the Air-STORM tool remains a useful resource for planning solar-powered air monitoring systems and can provide valuable insights into expected performance and failure risks.

The temperature bounds of 0 °C and 45 °C used in this study are based on commonly recommended operating limits for lithium-ion batteries and electronic components; however, these should not be interpreted as absolute cutoffs. Exceeding these thresholds may not immediately lead to system failure, as battery degradation and electronic performance are influenced by both temperature duration and cumulative exposure rather than instantaneous peaks. These limits are also likely somewhat conservative to account for potential safety margins and variations in manufacturer specifications. Recognizing that different applications may tolerate higher or lower risks, the Air-STORM tool allows users to adjust these temperature thresholds based on their specific deployment needs and risk tolerance, providing flexibility to accommodate a range of operating conditions.

The POWER data is available at 1° × 1° spatial resolution, which could affect the precision of the model in regions with high sub-grid heterogeneity. For cases requiring higher spatial resolution, the model could be adapted to use datasets such as MERRA-2 [30] (0.5° × 0.625° resolution) or ERA5 [31] (0.25° × 0.25° resolution), which offer higher spatial precision. However, POWER was chosen for this study as it is specifically tailored for solar energy applications, providing pre-processed data that reduces the computational burden when running the Air-STORM model. In special cases, the higher precision data or the collection of site-specific radiation data may be justifiable, but these will typically be special use cases.

Although the current implementation of Air-STORM uses rectangular enclosures, the model’s thermal predictions are primarily governed by surface area, material properties, and external environmental forcing, not geometry. For compact, sealed enclosures, internal temperatures are largely uniform due to low air heat capacity and dominant conductive and radiative exchange with external surfaces. Comparative calculations (Table S4) show that multiple enclosure shapes (e.g., boxes, cylinders, spheres) with similar surface area-to-volume ratios exhibit nearly identical thermal response characteristics. Although internal radiation between components was not explicitly modeled, measured temperature gradients within the enclosures were found to be small, resulting in negligible radiative redistribution. For the FDM framework used by Air-STORM, geometry would primarily influence mesh layout rather than net energy balance. As a result, enclosure shape would have minimal impact on predicted overheating, and no impact on battery depletion. A numerical comparison of enclosures of similar sizes is presented in the SI. Future versions of Air-STORM may allow user-defined shapes through equivalent surface area representations.

## 4. Conclusions

Expanding global air quality monitoring is essential for addressing knowledge gaps in air pollution exposure, particularly in remote and resource-limited regions. Solar-powered monitoring systems offer a promising solution but are susceptible to failures due to power shortages and temperature extremes. The Air-STORM tool developed for this study provides a predictive framework for evaluating the feasibility of deploying solar-powered monitors across diverse environmental conditions. By integrating historical meteorological data with a physics-based numerical heat transfer and energy balance model, Air-STORM enables users to assess the risk of temperature- and power-related failure before deployment.

Validation against experimental data demonstrated strong model agreement, with RMSE values ranging from 1.4 °C to 3.5 °C for internal temperature predictions. Simulations revealed that overheating and battery depletion present significant challenges in many global regions, emphasizing the importance of careful system design. Strategic mitigation measures, such as solar shielding, optimized panel orientation, and tailored battery sizing, can improve monitor reliability.

By providing an open-source, user-customizable tool, this work enables researchers and policymakers to make data-driven decisions for designing monitoring systems. While the current model is physics-based to support predictions with limited prior knowledge about the sampler or environment, future versions could integrate machine learning approaches as more deployment data become available. These data-driven methods may enhance predictive performance and enable adaptive system tuning in well-characterized regions. Ensuring reliable, long-term data collection in underserved regions will improve global air pollution assessments and contribute to more equitable environmental policy decisions.

## Figures and Tables

**Table 1:** Performance metrics comparing modeled and observed internal temperatures for four enclosures over one month. Observed temperatures were also compared to ambient air temperatures to establish a baseline and enable evaluation of Air-STORM’s performance relative to using ambient air alone. All units are in degrees Celsius. Performance metrics are presented in mean bias (MB), root mean squared error (RMSE), coefficient of determination (R^2^), and mean absolute error (MAE).

Simulated Temperature						Ambient Temperature				
Material	MB	RMSE	R^2^	MB of Daily Max	MAE of Daily Max	MB	RMSE	R^2^	MB of Daily Max	MAE of Daily Max
Aluminum	−1.89	3.50	0.87	−5.20	5.23	−3.43	5.59	0.68	−12.89	12.89
ABS	−1.10	2.62	0.92	0.00	2.37	−3.72	5.15	0.68	−9.58	9.75
Fiberglass	0.64	2.50	0.93	0.71	2.54	−1.78	4.21	0.79	−10.10	10.23
PVC	−0.35	1.42	0.97	1.20	2.24	−1.88	2.84	0.86	−6.48	6.60

## Data Availability

Data that support the validation of the model and tools of this study are available in a public repository [https://github.com/kyanshlipak/HT_monitor_model_public, URL accessed on 16 July 2025]. All other relevant data is available from the corresponding authors. The code developed for this study is publicly available at the same repository and may be used and modified with proper attribution. Commercial use for profit is not permitted.

## Supplementary Materials

The following supporting information can be downloaded at: https://www.mdpi.com/article/10.3390/s25154798/s1, Figure S1: High and low-temperature limits of a collection of common air pollution samplers (n = 55); Figure S2: New Delhi case study: average temperature exceedance rates (over 45 °C) modeled from 2014 to 2023 at every month of the year and hour of the day; Figure S3: New Delhi case study: average solar assembly failure rates (battery depleted) modeled from 2014 to 2023 at every month of the year and hour of the day; Figure S4 Heat map of monthly, diurnal failure rates of the predictive temperature and solar charging models in Montreal, Canada over 10 years (2014–2023). (Upper) Color darkness describes the total failure rate and color pigment describes whether the failure was induced by temperature drops (red) or batteries being empty (blue). (Lower left) temperature failure rates alone, (lower right) solar failure rates alone; Figure S5: Modeled vs experimental solar power and charging for a solar panel-fitted air quality monitor; Figure S6: Modeled vs experimental solar power and charging for a solar panel-fitted air quality monitor for 12 days; Figure S7: Conceptual schematic of tool including key inputs, data sources, and model outputs; Figure S8: Diagram of predictive heat transfer model methodology; Figure S9: 24-h validation of predictive heat transfer model with solar shield; Figure S10: Modeled internal temperature using POWER data with 10% and 90% bounds; Figure S11: Sensitivity analysis of duty cycling for a 4 day simulation; Figure S12: Sensitivity analysis of temperature mixing ratio (upper) and fan flow rate (cfm) (lower) for a 48-h simulation; Figure S13: Sensitivity analysis of predictive heat transfer model FDM node count for 48-h simulation; Figure S14: Sensitivity analysis of initial temperature input for 48-h simulation; Figure S15: Sensitivity analysis of heat transfer model wind speed for 48-h simulation; Figure S16: Sensitivity analysis of heat transfer model internal heat generation rate (in Watts) for 48-h simulation; Figure S17: Sensitivity analysis of heat transfer model air quality monitor enclosure emissivity for 48-h simulation; Figure S18: Sensitivity analysis of modeled shading for 48-h simulation; Figure S19: App Layout and graphical outputs for typical simulation; Figure S20: Average diurnal DNI and temperature profiles at the validation rooftop site from 15 July 2024–15 August 2024, during the validation testing period; Figure S21: Timeseries observed DNI and temperature profiles at the validation rooftop site from 15 July 2024–15 August 2024, during the validation testing period; Figure S22: Simulation example fully optimized solar assembly battery charge over time for the 2022 winter season in Evanston, Illinois; Table S1: Air pollution monitors used to establish reasonable bounds for temperature limits and estimated internal heat generation; Table S2: Empirical convection correlations; Table S3: Dominant convection; Table S4: Enclosure shape comparisons; Additional model development details, sensitivity analyses, validation data, and information on the samplers used in developing the tool. References [32,33,34,35,36,37,38,39,40,41,42,43,44,45,46] are cited in the Supplementary Materials.
