# Supplementary material for: Air-STORM: Informed Decision Making to Improve the Success of Solar-Powered Air Quality Samplers in Challenging Environments
Source: Sensors (Basel). 2025 Aug 4;25(15):4798. doi: 10.3390/s25154798 (PMC12349474; doi:10.3390/s25154798)
Supplement: Supplementary file 1 [file sensors-25-04798-s001.zip › sensors-3741903-supplementary.pdf]

Supporting Information

<u>Temperature Ratings and Regulation Technologies</u>

Although temperature-based failure is a recognized issue for any equipment outside, there is often a lack of appreciation of the large difference between air temperature and the temperatures found inside of enclosures exposed to solar radiation. The need to understand and be able to proactively plan for these high temperatures is what motivated the development of the predictive heat transfer tool.

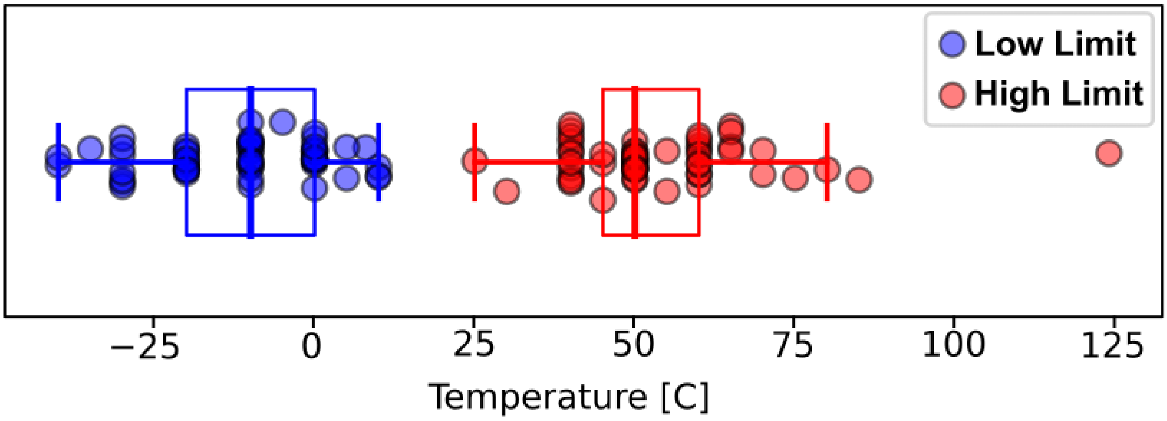

Figure S1: High and low-temperature limits of a collection of common air pollution samplers (n = 55 (References available in Table S1).

Figure S1 displays the distribution of temperature ratings for a range of 55 common air samplers. The median upper temperature limit found was 50 C, with a significant grouping having upper temperature limits at 40 C. Details of these results can be found in Table S1. These temperatures represent the manufacturers stated limits and may not account for temperature concerns associated with batteries or temperature related measurement bias. Table S1 also presented estimated heat generation from samplers where data is available. Nearly never do manufacturers report estimates of heat generated from instruments; estimates must be made based on electrical power usage. Conservation of energy requires that all energy into a sampler needs to go somewhere; for air pollution monitors, this is primary in the form of pump energy or heat. Although the efficiencies of air pumps, and other associated mechanical elements of a sampler, can vary greatly, electrical input power provides a valuable upper-bound estimate for heat generation.

Table S1: Air pollution monitors used to establish reasonable bounds for temperature limits and estimated internal heat generation.

Sampler	Lower Temperature Limit (C)	Upper Temperature Limit (C)	Estimated Heat Generation (watts)
2B Technologies - POM[1]	0	50	3
A.U.G. Signals Ltd - AirSENCE Standard AS400X[1,2]	-35	60	10
Aeroqual - S500[1]	-40	124	
Aeroqual S500-GSS[1]	0	40	
AethLabs - microAeth[1]	0	40	2.5
Air Quality Egg (v.2) - O3-SO2[1]	-20	40	2.5
Airly[1]	-30	40	
AirThinx IAQ[1]	-30	75	0.6
Airviz Inc. - Speck[1]	-10	65	2.5
Alphasense OPC-N2 / OPC-N3[1]	-10	50	0.875
Alphasense OPC-R2[1]	-10	40	
APIS[1,3]	-30	60	21
Applied Particle Technology (APT) - MINIMA[1]	0	65	
Blues Wireless - Airnote[1]	-10	45	
CairPol Cairsens[1]	-20	40	
Clarity Node[1,4]	-10	55	0.45
Davis Instruments - Airlink[1,5]	-10	60	0.5
Ecomesure - EcomSmart[1]	-20	40	
Edimax - AirBox[1]	0	50	
Elitech - Temtop LKC-1000S+[1]	0	50	
Elitech Temtop P20[1]	0	50	
Foobot[1]	0	50	
Hanvon-N1[1]	0	50	
Igienair Zaack AQI[1]	-10	40	
IQAir - AirVisual Outdoor[1]	-20	50	
Kaiterra Laser Egg 2+[1]	-10	60	
Liveable Cities[1]	-10	45	
Magnasci SRL - SMOGGIE-PM[1]	-20	65	
MetOne - ES-405[1]	0	50	4.2
Moji China - AirNut[1]	-20	70	
Oizom - Dustroid Pro V6[1,6]	-20	60	7
PAMAir - Airmazing PAS-OA-320-3G[1]	0	50	
Perkin Elmer - ELM[1]	-5	40	8
Piera Systems - Canree R1[1]	-10	60	

PM Monitor - iMonPM[1]	-40	85	7.5
Qingping - Air Monitor[1]	-10	50	
QuantAQ - MODULAIR-PM[1]	-20	60	1.25
Redspira[1]	-10	60	
SailBri Cooper SCI-901[1]	-30	55	100
Samyoung S&C - SY-DS-DK3[1]	10	65	
Sensirion - SGP40[1]	10	50	
Sensirion - SPS30[1]	-10	60	
Sensirion Nubo Air[1]	-10	50	
Strop de aer[1]	-20	80	
Tera Sensor - NextPM[1]	-20	70	
TSI - AirAssure[1]	10	30	
TSI - BlueSky[1,7]	-10	60	1
Vaisala - AQT410[1]	-30	50	0.5
Vaisala - AQT530[1]	-30	40	
Thermo Fisher Scientific 1405-F TEOM[8]	8	25	480
TSI DustTrak II Aerosol Monitor[9]	0	50	
Met One Instruments GT-521 (PM2.5, PM10)[10]	0	50	
Thermo Scientific - 5014i Beta Continuous Ambient Particulate Monitor[11]	-30	50	
Teledyne T500U[12]	5	40	80
TSI - Qtrak[13]	5	45	

New Delhi Case Study

Isolated solar and temperature diurnal/annual profile heatmaps for the New Delhi case study (main text Figure 7).

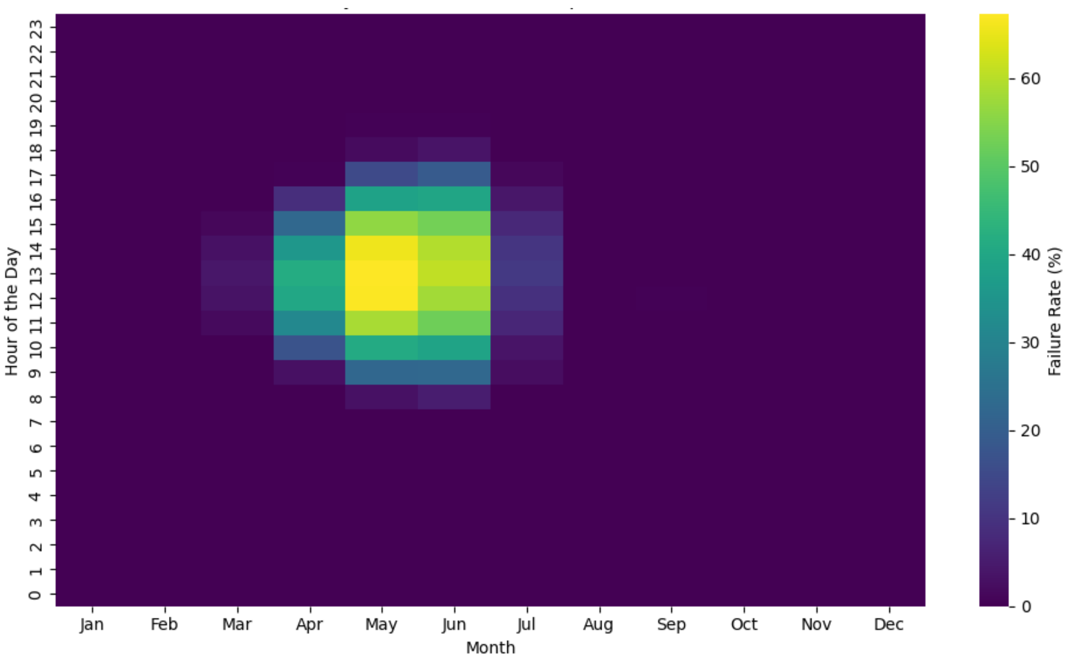

Figure S2: New Delhi case study: average temperature exceedance rates (over 45°C) modeled from 2014 to 2023 at every month of the year and hour of the day

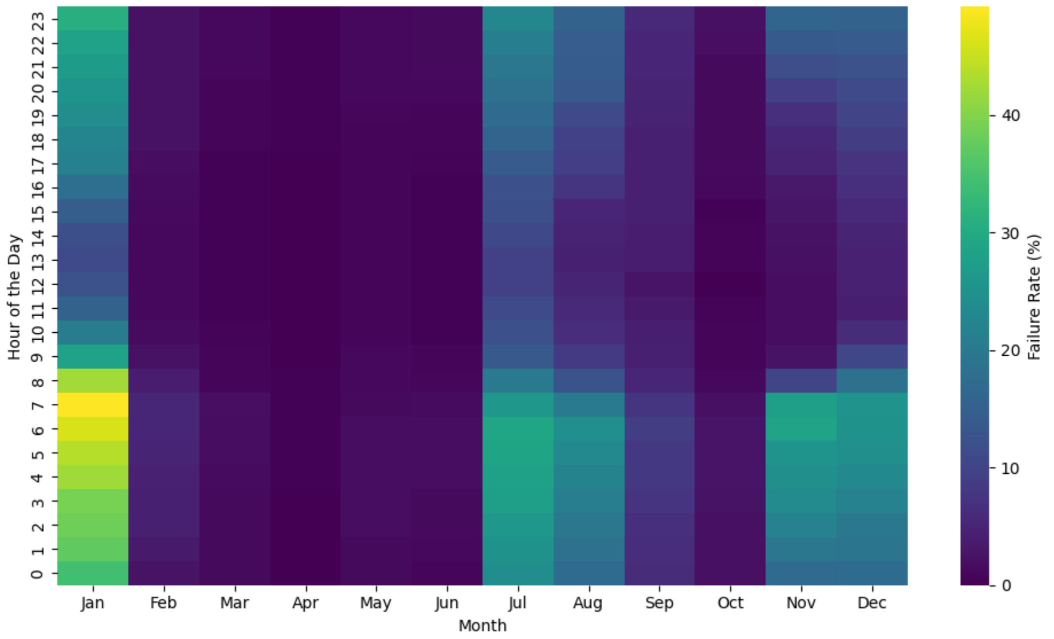

.

Figure S3: New Delhi case study: average solar assembly failure rates (battery depleted) modeled from 2014 to 2023 at every month of the year and hour of the day.

Figures S2 and S3 show the isolated solar and temperature failure rates, which are combined in Figure 7 of the main text. Figure S2 highlights how localized temperature failure rates are in this case study during the daylight hours of late spring and early summer. Despite warm temperatures during the summer months of July and August, cloudy and rainy weather prevents high solar potentials, reducing temperature exceedances. On the other hand, as seen in Figure S3, solar concerns are present in the low potential months of November, December, and January, and also during the monsoon season in July and August.

Montreal Case Study

A similar case study to the New Delhi analysis in the main text was done for a major city in a cold climate with both temperature and solar failure rates: Montreal. This analysis seeks to explore the utility of the tool's results over time to understand the interplay of the two failure modes in this coastal city.

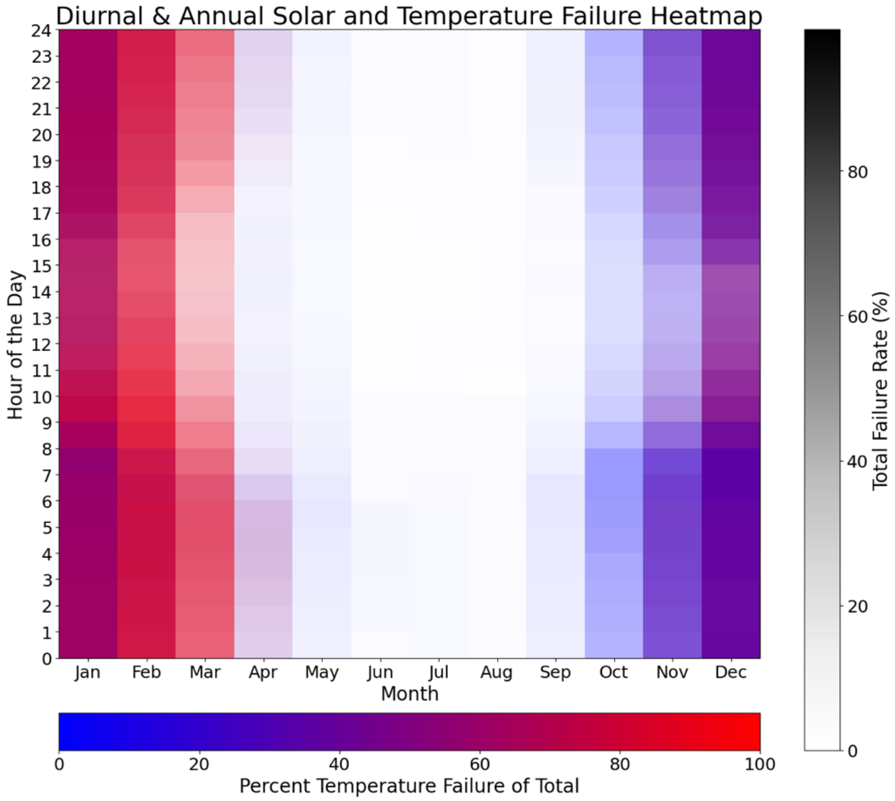

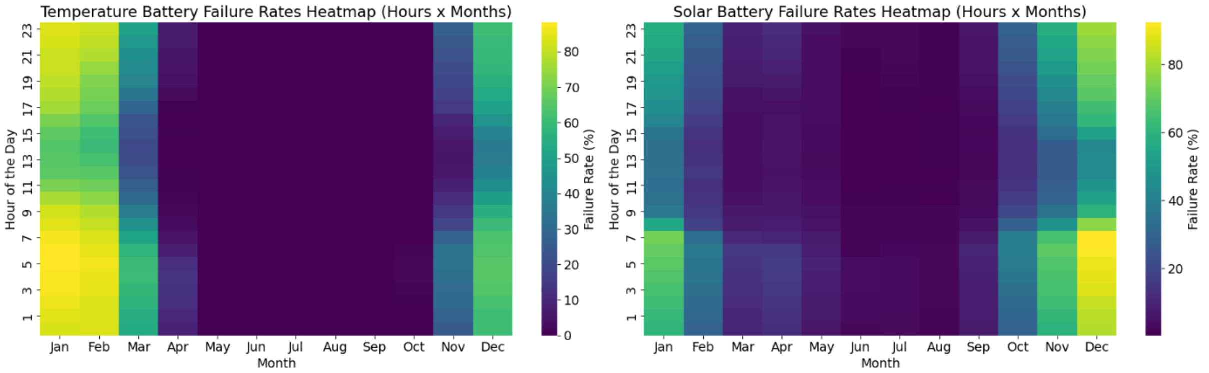

Figure S4 Heat map of monthly, diurnal failure rates of the predictive temperature and solar charging models in Montreal, Canada over 10 years (2014-2023). (Upper) Color darkness describes the total failure rate and color pigment describes whether the failure was induced by temperature drops (red) or batteries being empty (blue). (Lower left) temperature failure rates alone, (lower right) solar failure rates alone.

The heat map (Figure S4) shows monthly and diurnal failure rates predicted for a standard monitor, identical to those previously discussed, over a 10-year period (2014–2023) in a cold climate. Color intensity represents total failure rates, while color pigment differentiates between failure causes: red indicates simulated sampler temperatures were below freezing, and blue designates battery depletion due to insufficient solar charging. Both temperature and solar charging failures were concentrated in the colder months of the year and outside of peak daylight and solar potential hours (10:00-16:00), when both temperatures and solar charging rates rise.

A wider range of months exhibited extensive solar charging failures than the few months with frequent temperature failures due to freezing internal sampler temperatures. Solar failures occur in a distribution centered about the solstice, when days are the shortest and solar potential is the smallest. Other impacting factors on direct normal irradiance (DNI), such as cloud cover, did not appear to have significant seasonal trends. Temperature failures were concentrated in January and February, with simulated sample temperatures below 0ºC for more than 50% of the simulation period at each hour. Influenced by Montreal's location on the Lake of Two Mountains and Lake Saint Louis, temperatures do not drop as low during late Fall compared to during Winter months. Diurnally, both solar and temperature failures were lowest during daylight hours and highest just before dawn, when outdoor temperatures are lowest and samplers have depleted battery stores.

Solar Model Validation

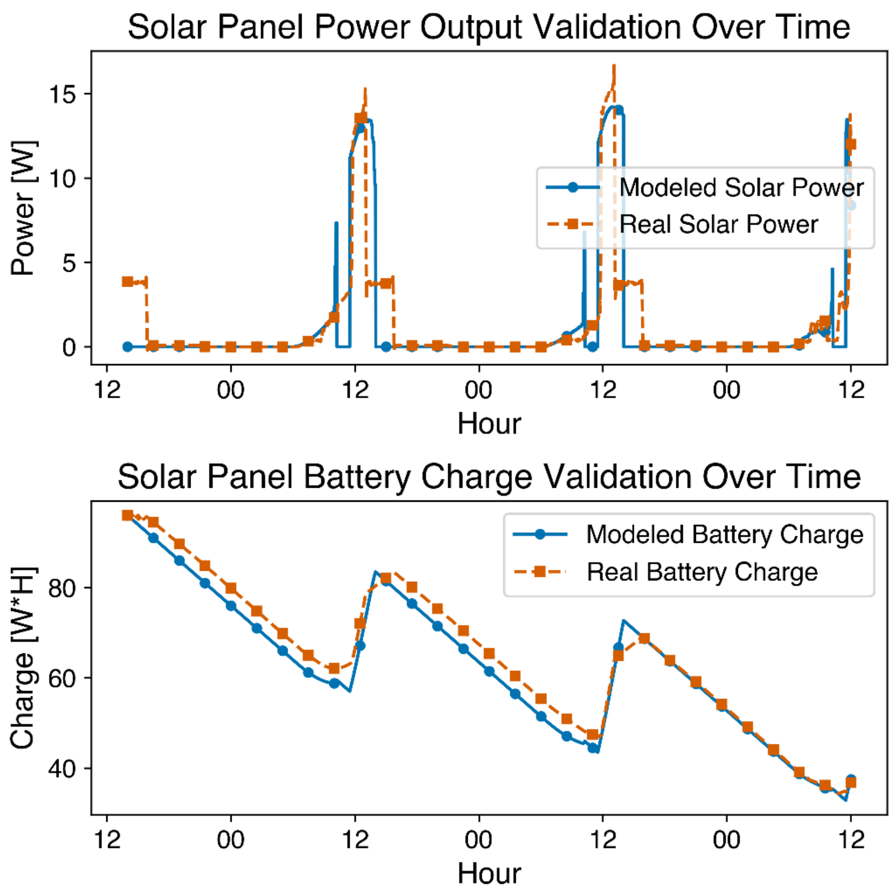

Figure S5: Modeled vs experimental solar power and charging for a solar panel-fitted air quality monitor.

The accuracy of the solar charging model was evaluated by comparing it to a sampler operating on a battery with solar charging co-located with the reference incident solar sensor. The model was highly accurate in replicating charging and discharging curves, with the worst performance when the solar panel was partially shaded. The 80 mm^2 reference sensor would often measure full sun or full shaded, whereas the larger panel would be in partial sunlight. Despite these minor discrepancies, the overall trends are closely aligned, as shown in Figure S5. It should be noted that prior to applying the model, solar panel performance was evaluated to establish charging efficiency.

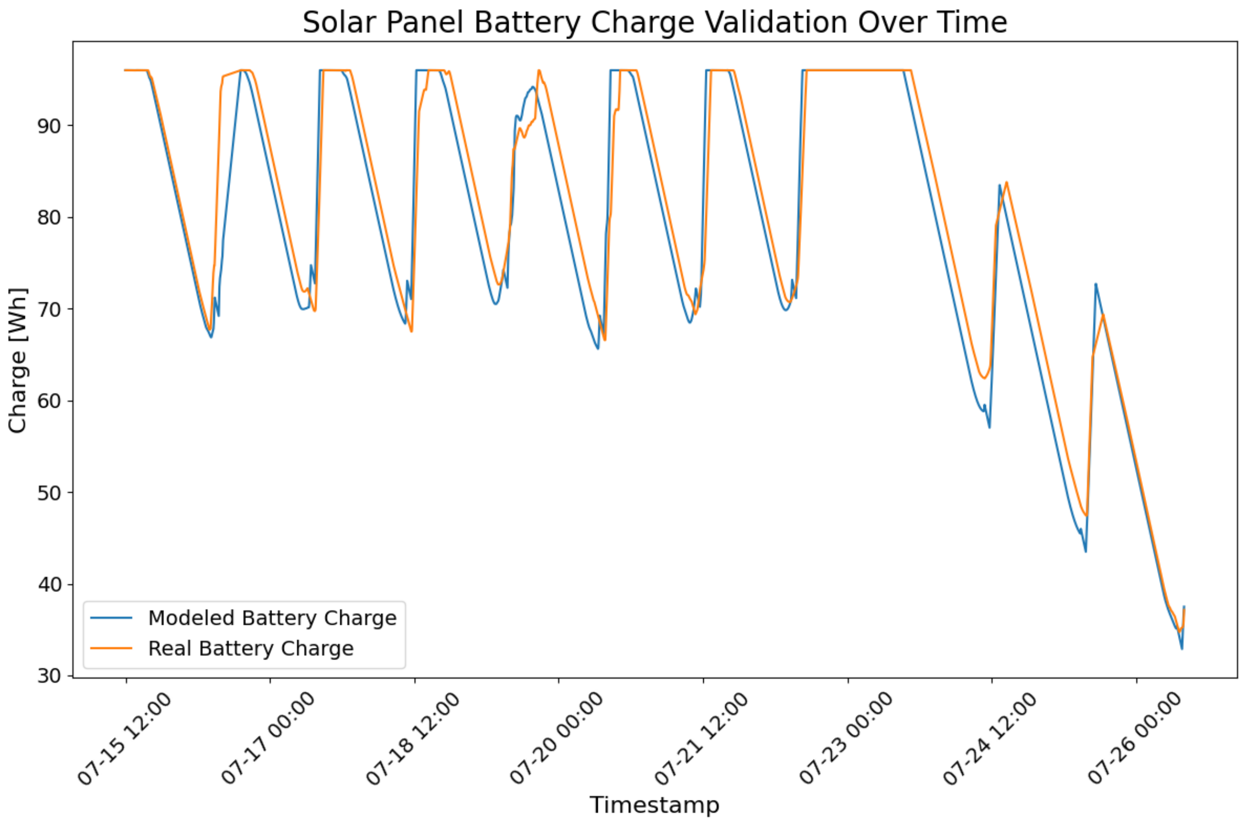

Figure S6: Modeled vs experimental solar power and charging for a solar panel-fitted air quality monitor for 12 days.

Temperature Model Validations

Predictive Heat Transfer Model Details

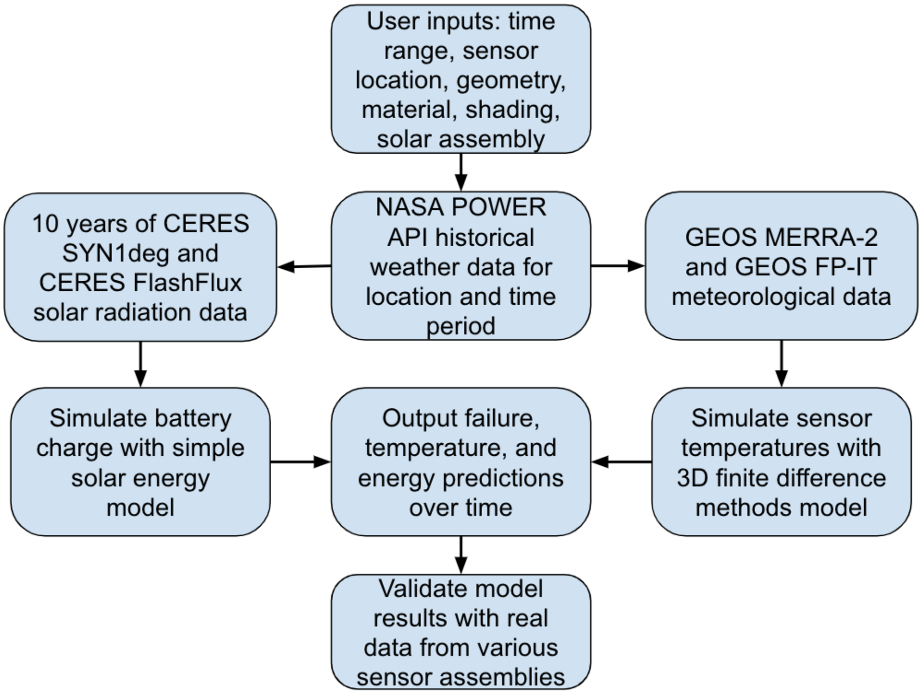

Figure S7: Conceptual schematic of tool including key inputs, data sources, and model outputs.

Figure S7 shows a diagram of the workflow of model development and usage. Model data inputs are discussed in further detail in the “Details on Model Inputs” section of the Supporting Information for this publication.

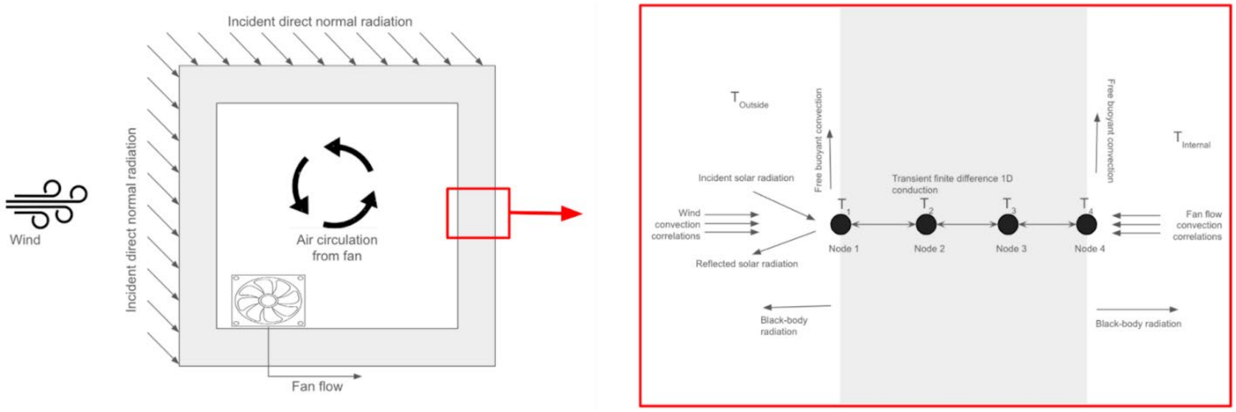

Figure S8: Diagram of predictive heat transfer model methodology

As discussed in the paper, the heat transfer model simulates each air quality monitor enclosure as six flat plates and assumes the direction of one-dimensional heat transfer through the walls of the enclosure to be normal to each face (Figure S8). Environmental variables considered are outdoor ambient temperature, direct normal radiation (DNI), solar elevation and azimuthal angle,

and 10m wind speed. Enclosure parameters include material properties, thickness, external dimensions, fan volumetric flow rating, fan heat generation, fan thermal switch temperature threshold, and air sample heat generation. Transient conduction is modeled using Finite Difference Methods (FDM) due to the simplicity of this method and the low computational expense relative to Finite Element Methods or Computation Fluid Dynamics. Below, the defining derivations of typical FDM are expanded upon in detail.

$$(1)\ \frac{\partial}{\partial x}\left(k\frac{\partial T}{\partial x}\right) = \rho C_p \frac{\partial T}{\partial t} \text{ and } \alpha = \frac{k}{\rho C_p} \text{ so } \alpha\frac{\partial^2 T}{\partial x^2} = \frac{\partial T}{\partial t}$$

$$(2)\ T_m^{p+1} = T_m^p + \frac{\alpha \Delta t}{(\Delta x)^2}\left(T_{m+1}^p - 2T_m^p + T_{m-1}^p\right)$$

FDM discretizes the one-dimensional thicknesses of each face of the monitor and applies the Heat Diffusion Equation (HDE) at each timestep to find the change in temperature at each node. The basic one-dimensional Heat Diffusion Equation (Equation 1) can be discretized into a nodal equivalent (Equation 2) by using a forward difference to approximate the time derivative $\frac{\partial T}{\partial t}$ and a centered difference to approximate the spatial second derivative $\frac{\partial^2 T}{\partial x^2}$.

$$(3)\ \text{Given } Fo = \frac{\alpha \Delta t}{(\Delta x)^2},\ T_m^{p+1} = (1 - 2Fo)T_m^p + Fo\left(T_{m+1}^p + T_{m-1}^p\right)$$

T_m^p the temperature at the node for the previous timestep. $1 - 2Fo \geq 0$ to maintain stability and prevent oscillation because a negative number would mean the previous temperature used would suddenly be negative, which is physically impossible. This is called the stability criterion of FDM, which in this case would be $Fo \leq \frac{1}{2}$, $\frac{\alpha \Delta t}{(\Delta x)^2} \leq \frac{1}{2}$, $\Delta t \leq \frac{(\Delta x)^2}{2\alpha}$.

$$(4)\ q_{convection} + q_{conduction} + q_{solar\,radiation} + q_{rad} = \rho C_p \partial V \frac{\partial T}{\partial t}$$

FDM at the surface nodes of the enclosure walls can be similarly derived from the equilibrium equation (Equation 4),

$$(5)\ hA\left(T_\infty - T_o^p\right) + \frac{kA}{\Delta x}\left(T_1^p - T_o^p\right) + GA\alpha_{abs} + \sigma\alpha_{abs}(T_\infty)^4 - \sigma\varepsilon\left(T_0^p\right)^4 = \frac{\rho C_p A \Delta x}{2}\left(\frac{T_0^{p+1} - T_0^p}{\Delta t}\right)$$

The discretization of Equation 4 yields Equation 5, where G is the incident solar radiation, and the two in the denominator of the right hand of the equation is because of the control volume of the outer node being half that of an inner node.

$$(6)\ T_0^{p+1} = T_0^p + \frac{2\Delta t}{\rho C_p \Delta x}\left[h\left(T_\infty - T_0^p\right) + \frac{k}{\Delta x}\left(T_1^p - T_0^p\right) + G\alpha_{abs} + \sigma\alpha_{abs}(T_\infty)^4 - \sigma\varepsilon\left(T_0^p\right)^4\right]$$

$$(7)\ T_0^{p+1} = T_0^p\left[1 - 2FoBi - 2Fo - 2Fo\frac{\Delta x}{k}\sigma\varepsilon\left(T_0^p\right)^3\right] + 2Fo\left[T_1^p + BiT_\infty + \frac{\Delta x}{k}G\alpha_{abs} + \frac{\Delta x}{k}\sigma\alpha_{abs}(T_\infty)^4\right]$$

Solving for the temperature of the surface node at the next timestep gives Equation 6. Substituting the dimensionless numbers $Fo = \frac{k\Delta t}{\rho C_p (\Delta x)^2}$ and $Bi = \frac{h\Delta x}{k}$ gives the simplified equation for the temperature of the outer surface node at the next timestep (Equation 7).

$$(8)\ n\Delta t \le \frac{(\Delta x)^2}{2\alpha\left[1 + Bi + \frac{\Delta x}{k}\sigma\varepsilon(T_{max})^3\right]}$$

The criterion of resolution for an external node (Equation 8) can be derived similarly to with an interior node: the multiplier of T_0^p must be greater than zero, where T_{max} is the maximum expected temperature of the outermost node, and a safety factor of $n = 1.2$ is used.

$$(9)\ G = DNI\left[\sin(\gamma_s)\cos(\beta) + \cos(\gamma_s)\sin(\beta)\cos\left(\alpha_s - a_p\right)\right]$$

Radiation to each face of the monitor was calculated using the DNI and solar elevation at each timestep (Equation 9). The model does not consider information about the azimuthal orientation of the enclosure, assuming the enclosure is oriented flat and upright with the solar azimuth modeled along the diagonal of the enclosure, the maximum solar irradiation case. Shading of the enclosure is modeled with the assumption that the enclosure is either fully in the sun or fully shaded, with no partial shading considered. Solar radiation to the enclosure under solar-shading conditions is modeled as 90% reductions in incident solar radiation to the top face and full solar radiation to each other face (Figure S9).

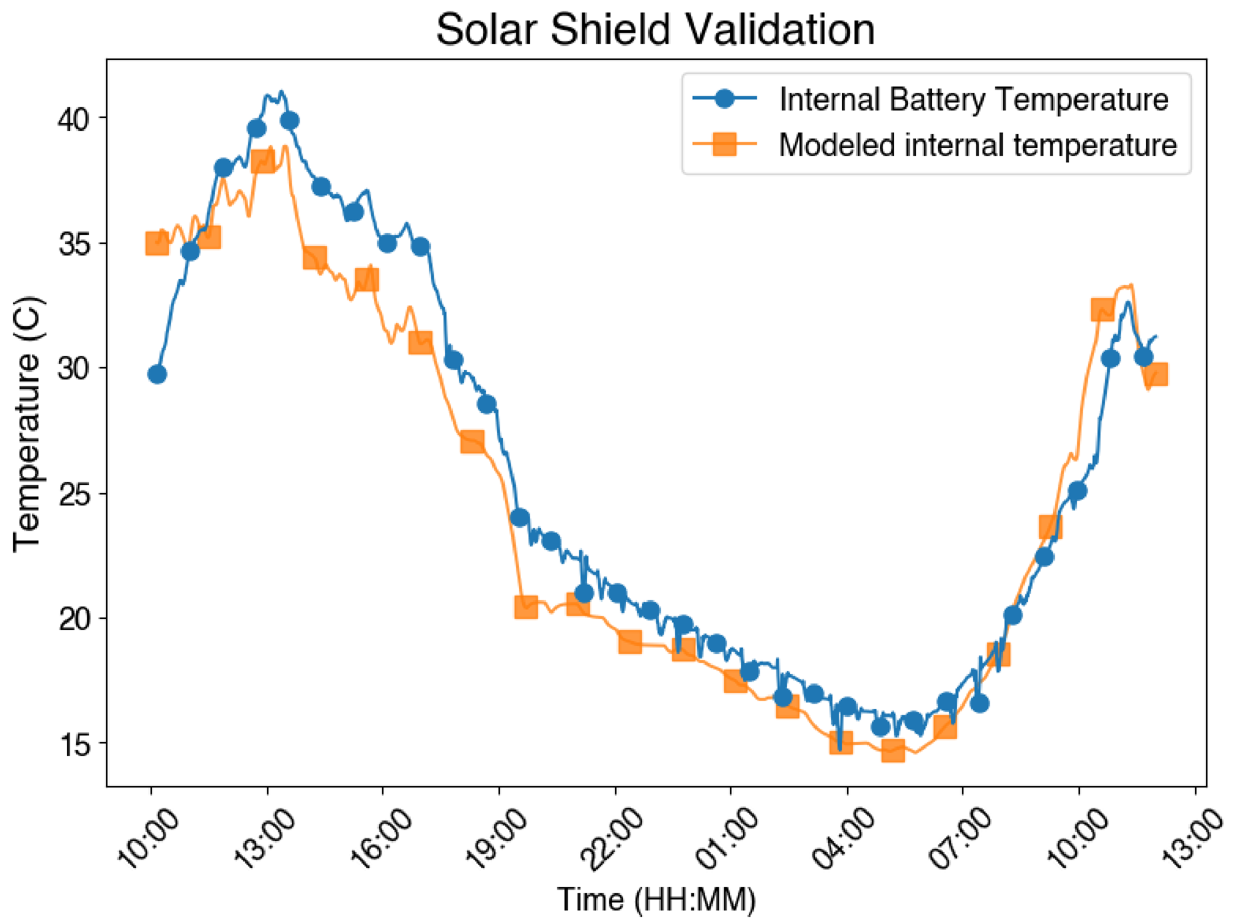

Figure S9: 24-hour validation of predictive heat transfer model with solar shield.

Table S2: Empirical convection correlations.

Description	Nusselt Number
Free convection vertical plate for laminar flow ($Ra < 10^9$)	$0.68 + \frac{0.67 Ra_L^{\frac{1}{4}}}{\left[1 + \left(\frac{0.492}{Pr}\right)^{\frac{9}{16}}\right]^{\frac{4}{9}}}$
Free convection vertical plate for all flow	$\left[0.825 + \frac{0.387 Ra_L^{\frac{1}{6}}}{\left[1 + \left(\frac{0.492}{Pr}\right)^{\frac{9}{16}}\right]^{\frac{8}{27}}}\right]^2$
Upper surface of hot plate of lower surface of cold plate ($10^4 < Ra < 10^7$)	$0.54 Ra_L^{\frac{1}{4}}$

Upper surface of hot plate of lower surface of cold plate ($10^7 < Ra < 10^{11}$)	$0.15Ra_L^{\frac{1}{3}}$
Lower surface of hot plate or upper surface of cold palte ($10^5 < Ra < 10^{10}$)	$0.27Ra_L^{\frac{1}{4}}$
Horizontal plate in parallel flow ($Re < 5 \cdot 10^5$)	$(\mathrm{Pr})^{\frac{1}{3}}Re_L^{0.8}$
Horizontal plate in parallel flow ($Re > 5 \cdot 10^5$)	$(\mathrm{Pr})^{\frac{1}{3}}\left(0.664Re_c^{0.5} + 0.037(Re_L^{0.8} - Re_c^{0.8})\right)$

Ra – Raleigh's Number
Nu – Nusselt Number
Ri – Richardson Number
Re – Reynold's Number
Pr – Prandtal's Number

Table S3: Dominant convection.

**Forced Convection**	**Free Convection**	**Mixed Convection**
Ri < 0.1	Ri > 10	0.1 <= Ri <= 10

Below, the convection correlations and methods are expanded upon in detail. Each face of the enclosure was modeled as a flat plate in parallel flow. The characteristic length was defined to be the geometric average of the width and length of the faces under forced convection and the area / perimeter for free convection correlations. Free convection was modeled with modern convection correlations based on the orientation of the face, position on enclosure, and temperature relative to the external temperature (see Table S2 for full equations and descriptions). Laminar and turbulent cases were distinguished by a critical Rayleigh's number of 10^9 for vertical faces and 10^7 for horizontal faces. The Richardson Number (Ri) was used to determine whether forced or free convection was dominant (Table S2), while the Nusselt number for the mixed convection case. Forced convection coefficients for the external faces of the enclosure were calculated using convection correlations assuming air flow velocity equal to the wind speed on each face (Figure S15). Forced convection coefficients on the inside of the box were assumed by calculating the air flow velocity as the fan volumetric flow divided by the average of each cross-sectional area of the enclosure. Placement of fan and direction of flow

were not implicitly assumed, so the convection coefficient was calculated to be the average of the convection heat transfer coefficients calculated for each of the three axial flow directions.

The temperature of the air within the enclosure is assumed to be equal to the average surface temperature of the internal faces of the enclosure (Figure 3 [left]). Battery surface temperature is modeled to be equal to the internal air temperature, which is consistent with experimental findings (Figure 3 [right]). Air flow due to internal fans was modeled to be 0.9 times the temperature of the air inside the enclosure + 0.1 times the temperature of the air outside the enclosure (Figure S12). Multiple experiments were conducted to test the mixing ratio of internal and outside air temperatures for use in convection correlations on the inside enclosure walls. Internal fans were both experimentally found to have relatively small impacts on internal air temperature due to the temperature of the air being dominated by the internal air temperatures. When measuring internal sampler temperatures with mounted mixing fans blowing air from outside the enclosure, most of the observed differences in experimental observed internal temperatures with a fan could be attributed to the increased heat generation associated with the electronics operating the fan. Bulk airflow on the inside of the sampler is generally an ineffective method of cooling the monitor and battery because air has a limited specific heat capacity relative to the higher thermal masses of typical enclosure materials and is thus unable to transfer sufficient heat.

To reduce the computational expense of the FDM model when simulating long periods of time or high temporal resolutions, periodic duty cycling of the model was performed by linearly approximating during “off-cycles", while full model fidelity was maintained during “on-cycles”. Typical duty cycling was optimized to a 1:3 ratio of FDM simulated time periods to linearly approximated time periods in order to minimize computational time while keeping the FDM stable (Figure S11).

Solar Energy Model Details

As discussed in the main text, the battery charge of air quality monitors with solar units is modeled by calculating the solar power input and total power output at each timestep. Solar power input to the battery is estimated by multiplying the total incident solar radiation by the solar panel efficiency. The solar azimuth and elevation angles are modeled using the Ephem orbital model for a given time and location. The battery is assumed not to charge above its rated capacity or discharge below zero charge.

Details on Model Inputs

Meteorological and solar radiation data for the predictive heat transfer and solar energy models is derived from the NASA Prediction of Worldwide Energy Resources (POWER) database via the API service. The POWER database provides geospatially continuous solar and meteorological datasets from 2001 to real-time at an hourly resolution.

Historical meteorological data is provided at a ½° x ⅝° latitude/longitude resolution. Data for up to one month behind real time is derived from the Modern-Era Retrospective Analysis for Research and Applications version 2 (MERRA-2) reanalysis mode, the most recent atmospheric reanalysis built by the NASA Global Modeling and Assimilation Office using data from the Goddard Earth Observing System (GEOS) model. POWER meteorological data within one month of real time is produced by the GEOS Forward Processing for Instrument Teams (GEOS FP-IT) and provides near-real time reanalysis capabilities.

Historical solar radiation data is provided at a 1° x 1° latitude/longitude grid. Data for up to a few months within real time is produced using NASA's Clouds and the Earth's Radiant Energy Synoptic (CERES SYN1deg [14]) model., The CERES SYN1deg model produces solar radiation and cloud cover data using input information from the Terra and Aqua Moderate Resolution Imaging Spectroradiometer (MODIS) combined with meteorological data from GEOS. NASA's Fast Longwave and Shortwave Flux (CERES FLASHFlux [15]) project is used to generate solar radiation data from the end of CERES SYN1deg to approximately one week behind real-time.

Validation of Bounding Variation

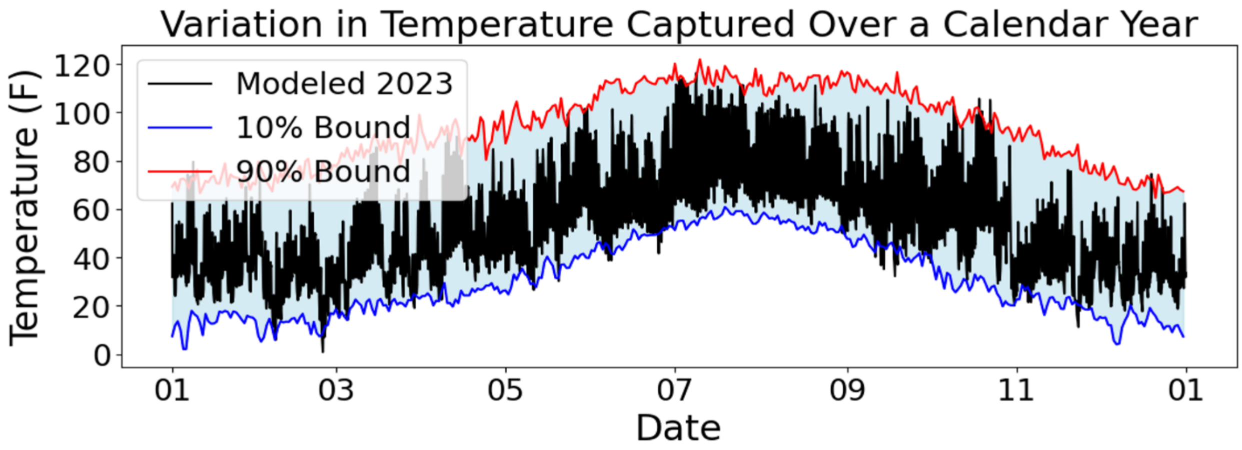

Figure S10: Modeled internal temperature using POWER data with 10% and 90% bounds.

Figure S10 shows an example usage of historical data to bound variability that can be used in the web-tool of the Air-STORM model. The 10% and 90% bounds captured 97% of modeled daily maximum and 87% of daily minimum internal enclosure temperatures in Fort Collins over

five randomly selected years from 2001 to 2023. Absolute maximum and minimum temperatures of each year were captured by the 10% and 90% bounds, suggesting extreme weather events can be bounded by the 10% and 90% NASA POWER hourly data. Figure S10 shows an example validation of the 10% and 90% bounds using meteorological data from 2023 while modelling Fort Collins.

Model Sensitivity Analyses

Validation of Reducing Complexity

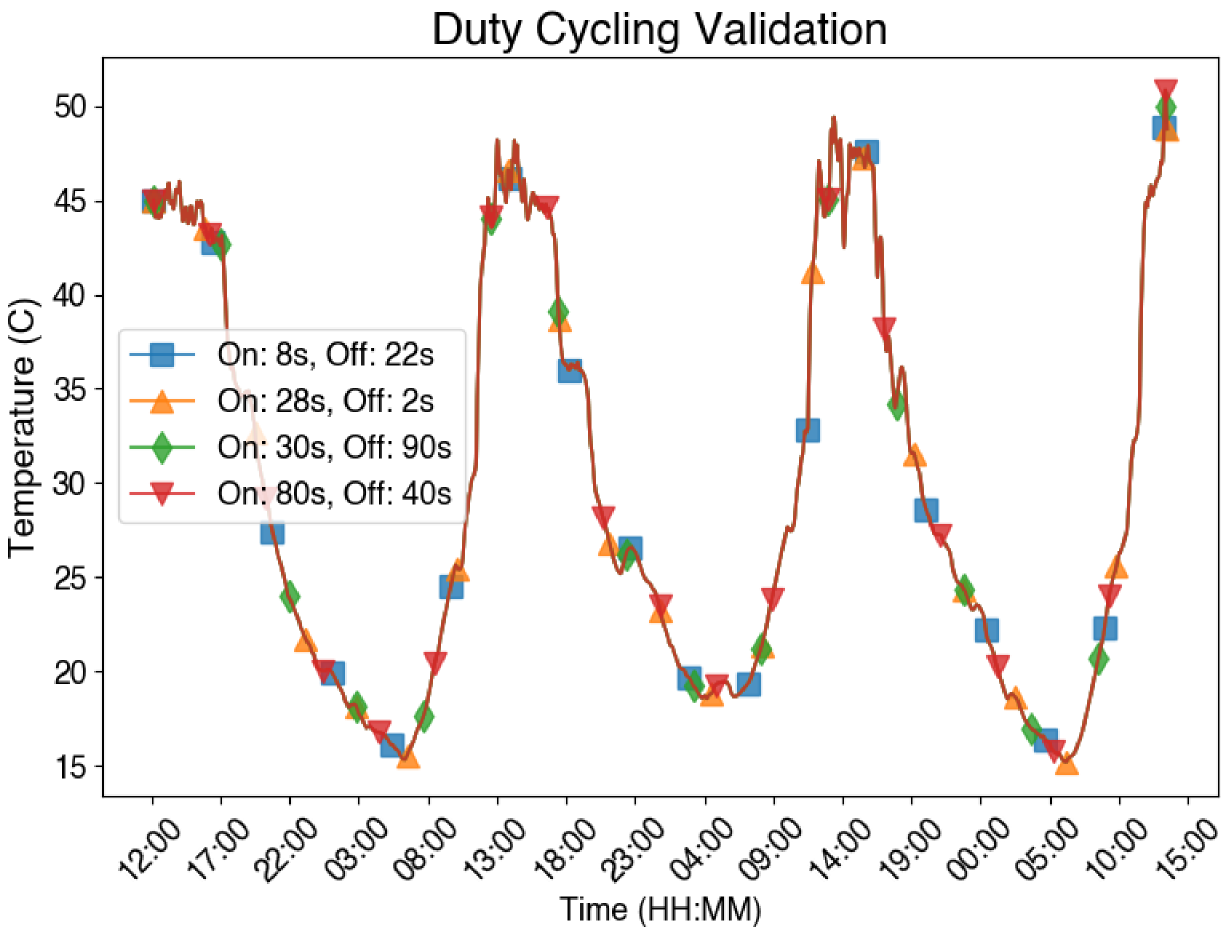

Figure S11: Sensitivity analysis of duty cycling for a 4 day simulation.

Duty cycling the model to reduce the duration of simulated finite difference methods (FDM) modeling allowed for the reduction of computational expense without statistically significant reductions ($p = 1.000$) in modeling accuracy. During “off cycles”, both environmental variables and modeled temperatures were linearly approximated, while during on cycles, the full fidelity FDM model was used. A ratio of 3:1 for off-cycle:on-cycle with 90:30 second simulation time

duty cycling was found to maximize the reduction of computational expense while maintaining model stability.

Model error (RMSE) and correlation coefficients (r) were identical between different duty cycle parameters in the sensitivity analysis above (Figure S11) to 5+ decimal points, indicating no significant difference between modeling with the different selected duty cycling parameters.

Fan Sensitivity

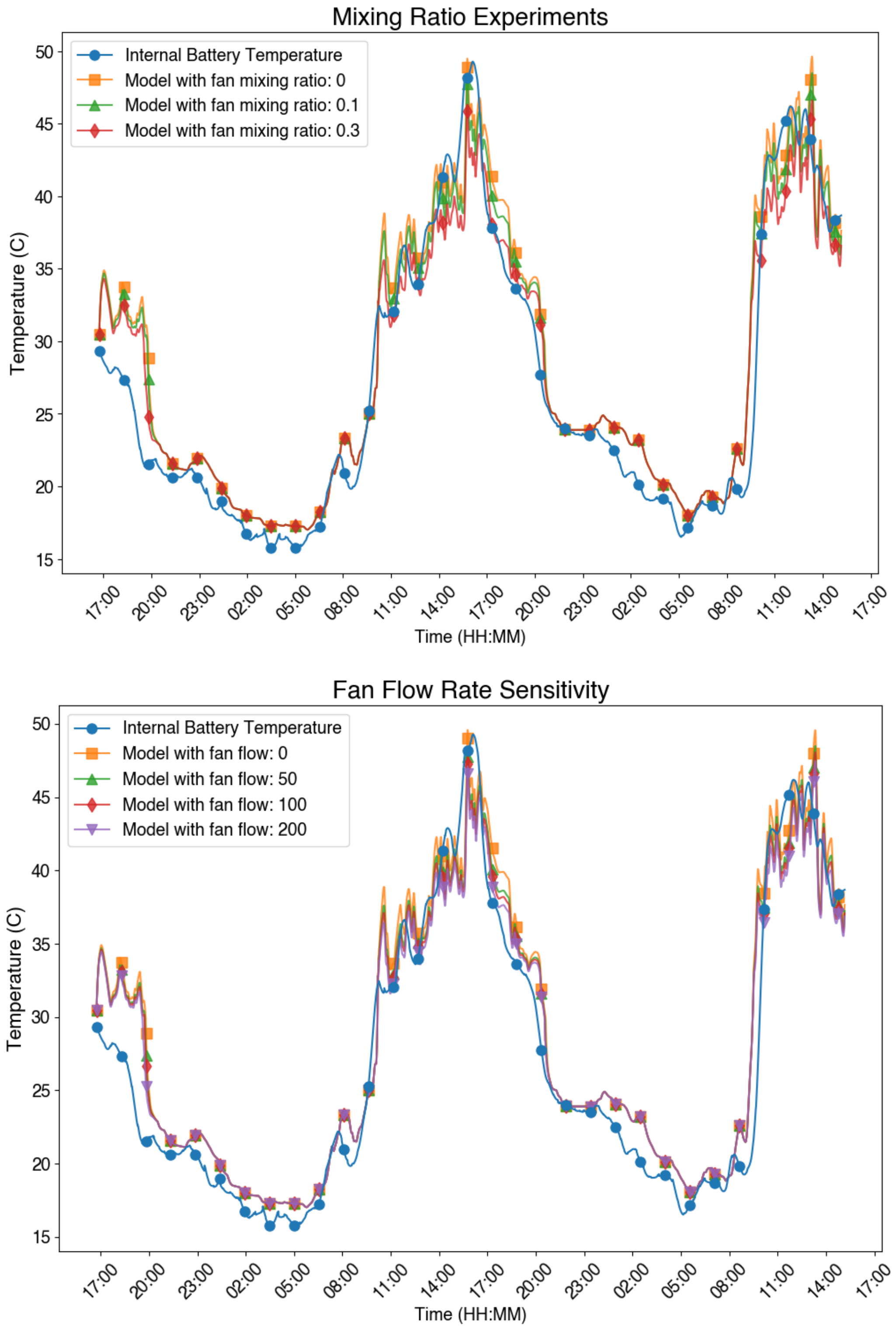

Figure S12: Sensitivity analysis of temperature mixing ratio (upper) and fan flow rate (cfm) (lower) for a 48-hour simulation.

Experiments were conducted with a fan of 50cfm and 9W heat generation and a 30C thermal switch. Model sensitivities compared simulated results with identical parameters (i.e. 30C fan threshold, 9W heat generation).

Modeling air flow blown by a fan from the external environment of the enclosure as equal in temperature 0.9 times the internal air temperature of the enclosure plus 0.1 times the outside air temperature was found to yield the most accurate results. Model results of fan speed sensitivity analyses demonstrate small cooling effects of fans with fan flow rates surpassing 50cfm for a typical enclosure (20cm x 15cm x 10cm) (Figure S12).

Fan use is generally not recommended by this study for preventing the overheating of air sampler enclosures. In each outdoor experiment conducted as a part of this study, the heat generation of the fan assembly was significantly larger than any cooling contributions.

Number of Nodes Sensitivity

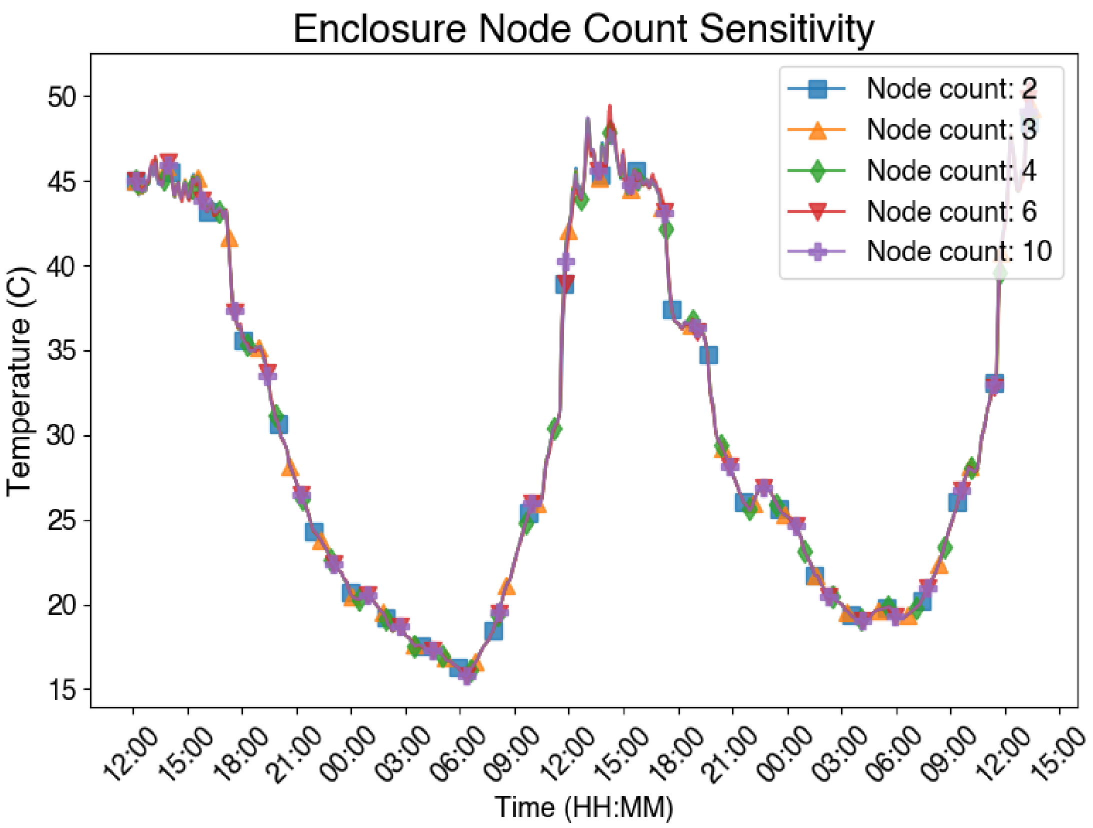

Figure S13: Sensitivity analysis of predictive heat transfer model FDM node count for 48-hour simulation.

A sensitivity analysis of the number of nodes modeled in the FDM heat transfer simulation was conducted, identifying no statistically significant differences between modeling with any number of nodes greater than 2 (Figure S13) while modeling thin enclosures (wall thickness < 1cm).

Initial Temperature Sensitivity

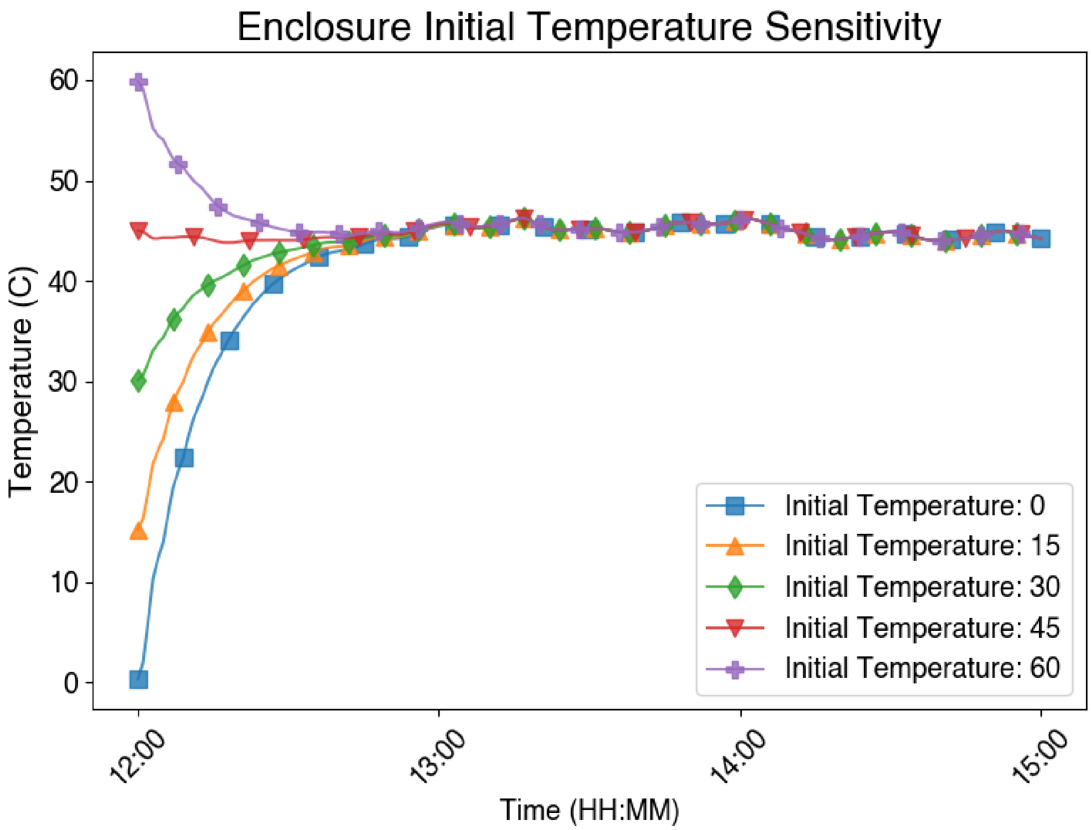

Figure S14: Sensitivity analysis of initial temperature input for 48-hour simulation.

The initial starting temperature was found to be insignificant when modeling for long periods of time, with temperatures converging within the first 3 hours of simulation time (Figure S14).

Wind Speed Sensitivity

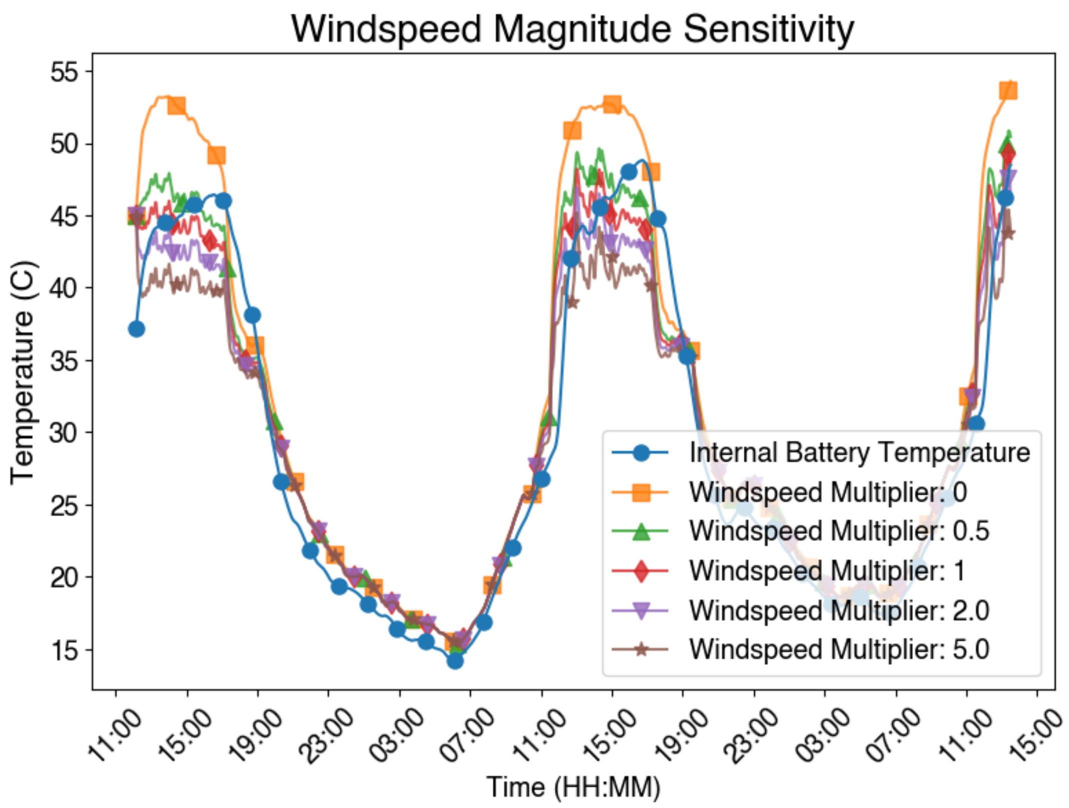

Figure S15: Sensitivity analysis of heat transfer model wind speed for 48-hour simulation.

A sensitivity analysis was conducted by multiplying weather station windspeed measurements used as inputs for the predictive heat transfer model by a fixed factor. Wind speed has a significant impact on modeled temperatures, particularly noticeable when comparing the zero-wind-speed control case (factor = zero) to the typical windspeed case (factor = one). The sensitivity reveals asymptotic behavior of modeled internal temperature as wind speeds increase and internal enclosure temperature approaches external temperature (Figure S15).

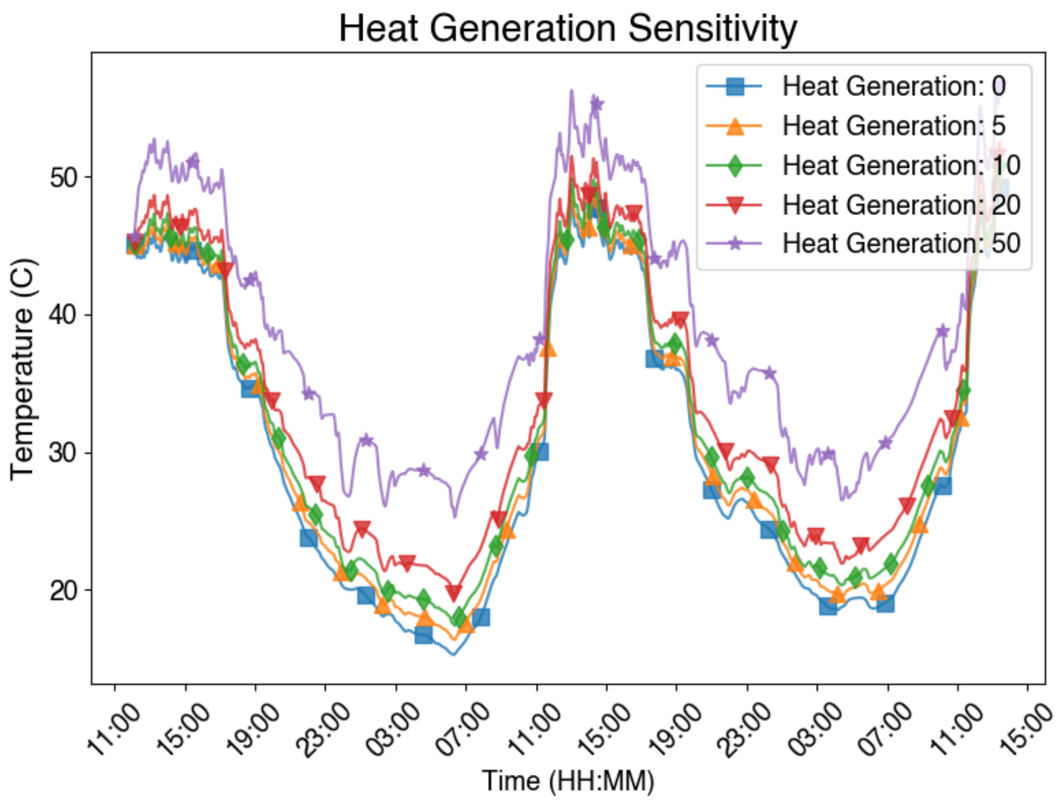

Figure S16: Sensitivity analysis of heat transfer model internal heat generation rate (in Watts) for 48-hour simulation.

Heat transfer model internal air temperature predictions are sensitive to internal heat generation. Figure S16 compares observed internal monitor air temperatures (with 2W heat generation) to simulated temperatures with varying modeled constant internal heat generations. Intuitively, increased internal heat generation leads to increases in simulated temperatures.

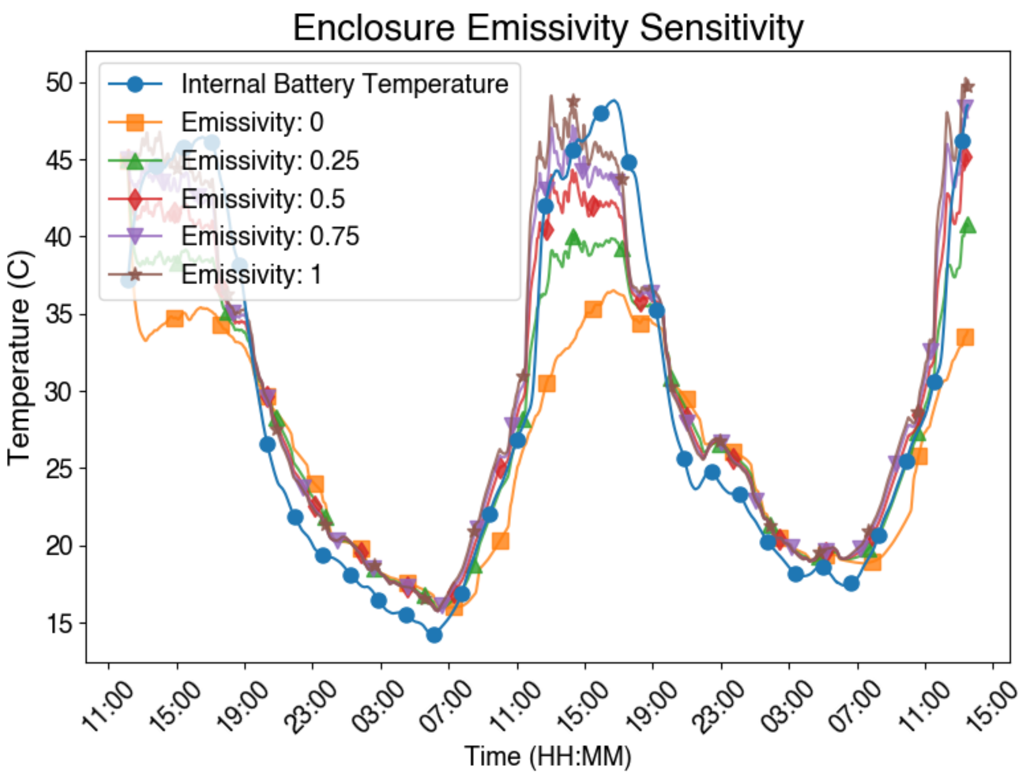

Figure S17: Sensitivity analysis of heat transfer model air quality monitor enclosure emissivity for 48-hour simulation.

Figure S17 compares observed monitor internal air temperatures to simulated temperatures with varied modeled enclosure material emissivity. Absorptivity is assumed to be equal to emissivity and emissivity is assumed to be constant with respect to temperature. Higher emissivity led to increases in modeled internal temperatures due to increases in absorbed solar radiation.

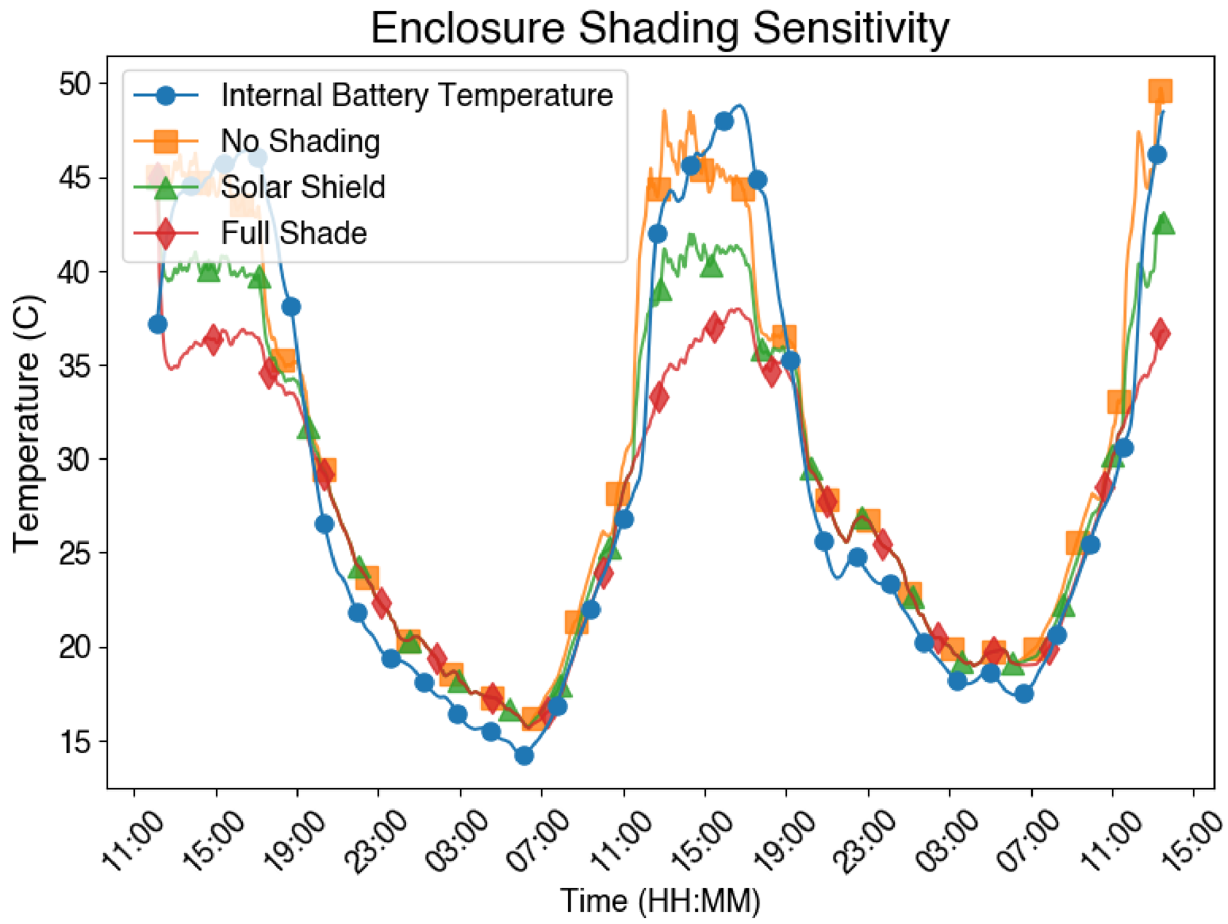

Figure S18: Sensitivity analysis of modeled shading for 48-hour simulation.

Figure S18 compares the three modeled cases described in the main text: an air quality monitor in full shade, an air quality monitor equipped with a solar shield and experiencing indirect solar radiation and reradiation from the shield, and an air quality monitor with no shading to observed internal temperatures for an unshaded enclosure. These days were sunny and hot, resulting in full shading reducing simulated temperatures by 10ºC.

<u>Impact of Enclosure Shape on Internal Heat Transfer</u>

To evaluate whether enclosure shape significantly affects thermal performance in small, solar-powered sensor systems, we numerically compared five hypothetical enclosure geometries: cube, rectangular box, short cylinder, tall cylinder, and sphere. Each hypothetical enclosure had an approximate internal volume of ~1000 cm^3. The comparison

focused on surface-area-to-volume (SA/V) ratio and internal radiative flux, shown in Table S3.

Surface-Area-to-Volume Ratio

SA/V ratios ranged from 0.48 to 0.66 cm^{-1} across the shapes, suggesting broadly similar thermal responsiveness to external environmental conditions. In small enclosures, SA/V will be a dominant factor in determining how quickly a device heats or cools, irrespective of exact shape.

Internal Radiative Flux Estimation

To quantify internal radiative heat redistribution, we estimated radiative flux between internal surfaces assuming a small but realistic temperature difference of 0.5 °C. Radiative heat exchange was calculated using the Stefan–Boltzmann law:

$$q\_rad = \varepsilon\sigma(T\_hot^4 - T\_cold^4)$$

Where $\varepsilon = 0.9$, $\sigma = 5.67 \times 10^{-8}$ W/m^2K^4, T_hot = 313.15 K, and T_cold = 312.65 K.

We conservatively assumed that only half the enclosure's internal surface area participates in radiative exchange, representing opposing "hot" and "cold" surfaces. This is consistent with standard enclosure modeling practice when temperature gradients are small and geometry is symmetric.

The resulting internal radiative fluxes ranged from 0.07 to 0.09 W across all geometries, reinforcing that geometry has a minor effect on the net energy balance under typical operating conditions. The analysis help reaffirm that enclosure shape will have a minimal influence on overall heat transfer when surface area, material, and environmental conditions are help constant.

Table S4: Enclosure shape comparisons.

Shape	Surface Area (cm^2)	Volume (cm^3)	SA/V Ratio (cm^{-1})	Internal Radiative Flux (W)
Cube	600.0	1000.0	0.60	0.09
Rectangular Box	592.0	960.0	0.62	0.09
Short Cylinder	471.2	785.4	0.60	0.07
Tall Cylinder	525.3	791.8	0.66	0.08
Sphere	483.1	998.3	0.48	0.08

Graphical Application

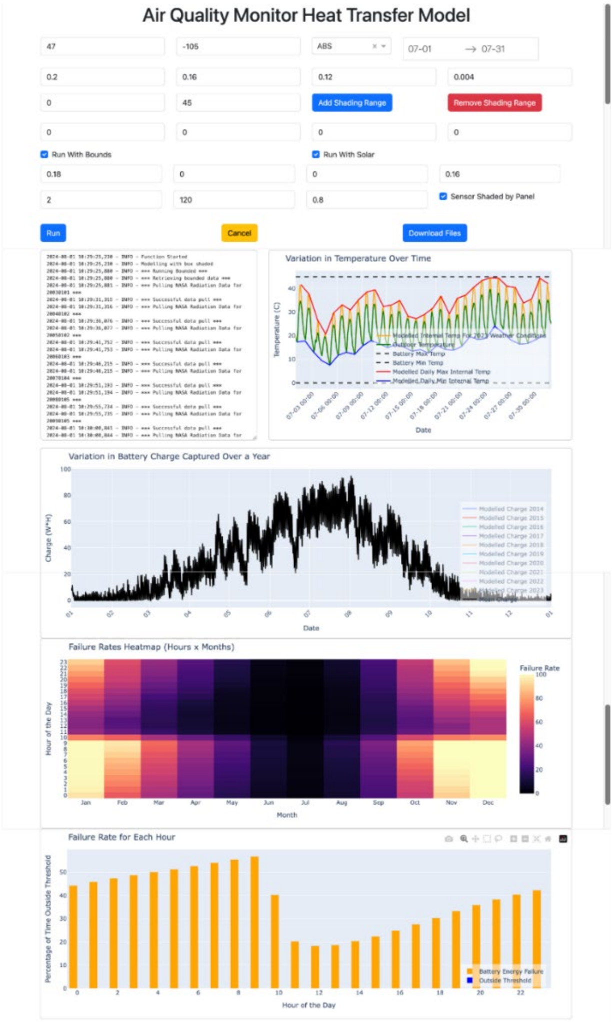

Figure S19: App Layout and graphical outputs for typical simulation.

An application with graphical user interface (Figure S19) was built to allow users to use the heat transfer and solar radiation models to easily model air pollutant sensors in any given location with limited programming knowledge or Python experience. The app is built using Plotly-Dash/Flask and can handle multiple users and instances simultaneously. The web app allows users to model up to a month of temperature predictions and ten years of solar charging predictions. Data products produced from the model include detailed results for estimated temperature and solar-charging failure rates, interactive plots of temperature and solar charge over time, and heat maps and plots of failure rates over time.

Validation Setup

Fiberglass large enclosure (16 x16x8 inches)

smallest enclsoure, aluminum (3x3x3 inches)

Purple Air Classic Sensor (Schedule 40 3 Inch PVC Endcap)

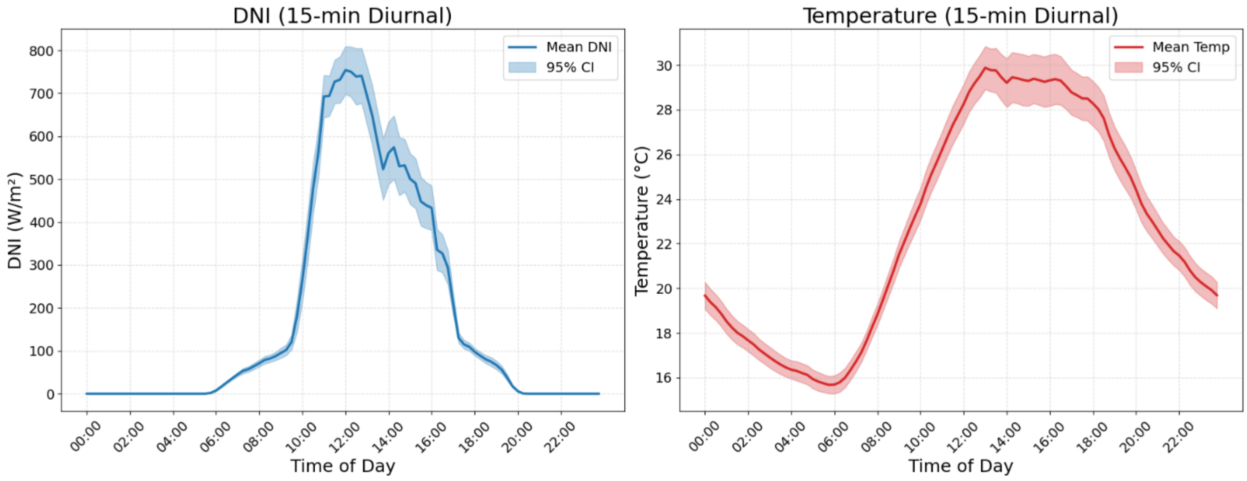

Figure S20: Average diurnal DNI and temperature profiles at the validation rooftop site from 07/15/2024 - 08/15/2024, during the validation testing period.

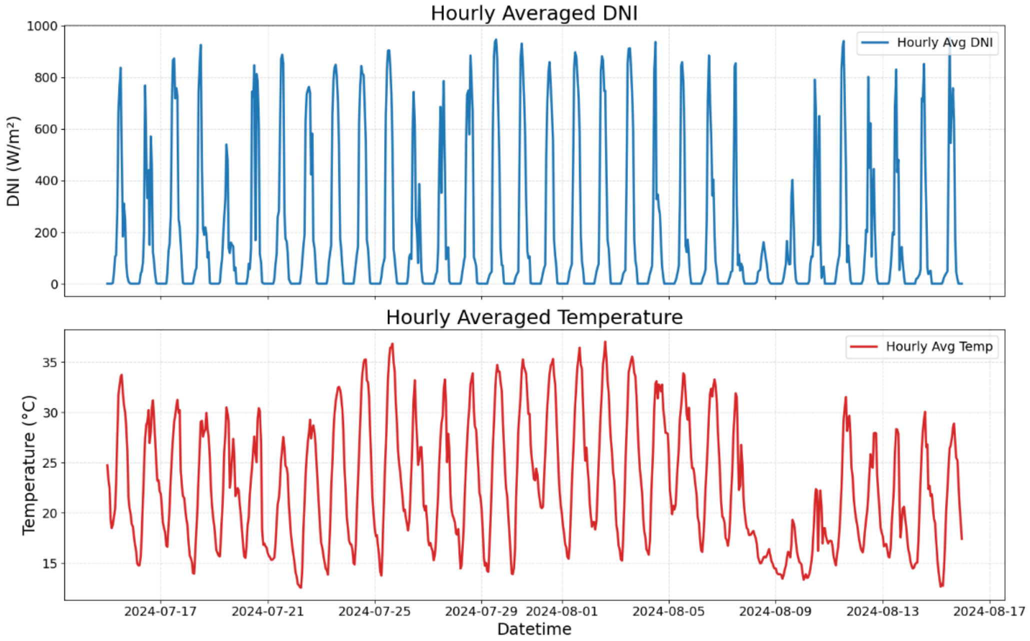

Figure S21: Timeseries observed DNI and temperature profiles at the validation rooftop site from 07/15/2024 - 08/15/2024, during the validation testing period.

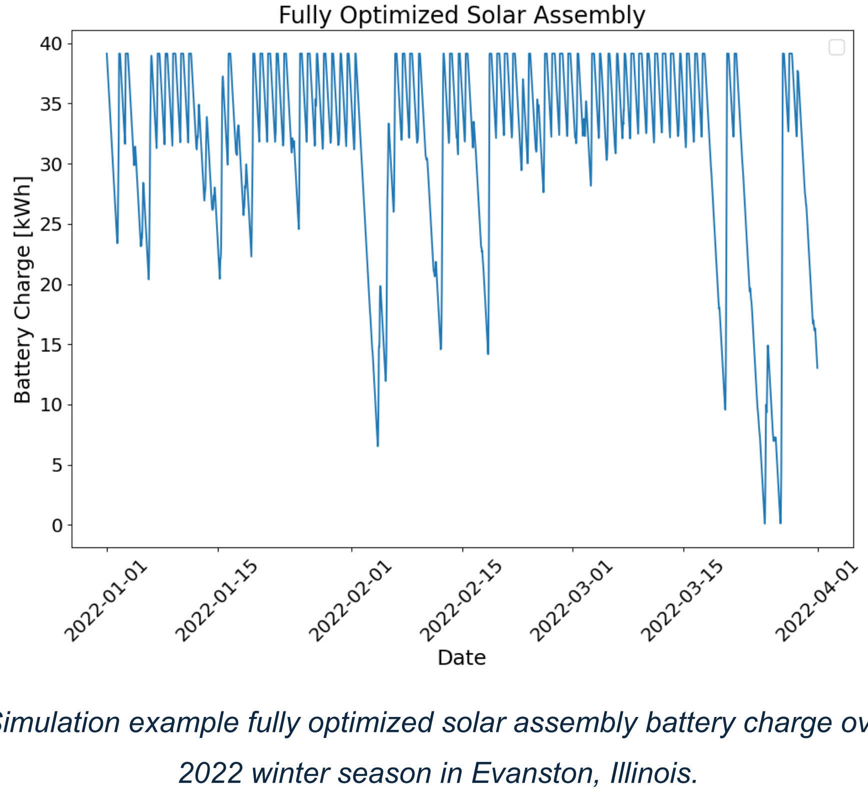

Figure S22: Simulation example fully optimized solar assembly battery charge over time for the 2022 winter season in Evanston, Illinois.

Figure S22 provides an example usage case of the solar simulation tool for an extremely high-power device in a challenging environment. Evanston, Illinois was chosen for this example due to its cold and dark winters and author familiarity. The battery size, solar panel size, and panel orientation were optimized to maximize winter charging while providing 500W of power without ever fully depleting, a realistic design case and usage of the tool. The solar panel area modeled was 74.6 m^2 with a 16% charging efficiency, and the total battery capacity was optimized to 48.9kWh of total capacity with 80% efficiency of effective storage capacity.

Support Information References

1. District SCAQM. Air Quality Sensor Performance Evaluation Center - Sensor List [Available from: http://www.aqmd.gov/aq-spec/sensors.
2. AirSENCe. AirSENCE [Available from: https://airsence.com/.
3. AQ A. Product Information [Available from: https://www.apis-aq.com/product-information/#specs.
4. Clarity. Clarity Node-S [Available from: https://www.clarity.io/products/clarity-node-s.
5. Instruments D. AirLink AIR Quality Monitor by Davis Instruments [Available from: https://www.davisinstruments.com/products/airlink-professional-air-quality-monitor.
6. Oizom. Online Dust Monitoring System [Available from: https://oizom.com/product/dust-monitoring-system-dustroid-pro/.
7. TSI. BlueSky Air Quality Monitor 8145 [Available from: https://tsi.com/products/environmental-air-monitors/bluesky-air-quality-monitor-8145]
8. Scientific T. 1405-F TEOM™ Continuous Ambient Air Monitor [Available from: https://www.thermofisher.com/order/catalog/product/TEOM1405F?SID=srch-srp-TEOM1405F.
9. TSI. DustTrak™ II Aerosol Monitor 8532 [Available from: https://tsi.com/products/aerosol-and-dust-monitors/aerosol-and-dust-monitors/dusttrak%E2%84%A2-ii-aerosol-monitor-8532/.
10. Met One Instruments I. GT-521S Handheld Particle Counter [Available from: https://metone.com/wp-content/uploads/2019/10/GT-521S.pdf.
11. Scientific T. 5014i Beta Continuous Ambient Particulate Monitor [Available from: https://www.thermofisher.com/order/catalog/product/5014I?SID=srch-srp-5014I.
12. API T. Model T500U [Available from: https://www.teledyne-api.com/products/nitrogen-compound-instruments/t500u.
13. TSI. Q-Trak Indoor Air Quality Monitor 7575 [Available from: https://tsi.com/products/indoor-air-quality-meters-instruments/indoor-air-quality-meters/q-trak-indoor-air-quality-monitor-7575/.
14. Earthdata N. CERES and GEO-Enhanced TOA [Available from: https://asdc.larc.nasa.gov/project/CERES/CER_SYN1deg-1Hour_Terra-Aqua-MODIS_Edition4A.
15. Earthdata N. Fast Longwave And SHortwave Radiative Fluxe [2024]. Available from: https://asdc.larc.nasa.gov/project/FLASHFLUX.
